# Piezo1 Upregulation in Monocyte‐Derived Macrophages Impairs Post‐Myocardial Infarction Cardiac Repair via Defective Efferocytosis and Enhanced Ferroptosis

**DOI:** 10.1002/advs.202510991

**Published:** 2025-11-10

**Authors:** Lu Peng, Yunlong Xia, Huishou Zhao, Yongzhen Guo, Xiaoming Xu, Xue Han, Shiyue Wang, Fengyue Ding, Quanchi Liu, Congye Li, Yuan He, Zhaoyi Luo, Qiaojuan Wang, Xing Zhang, Feng Gao, Yajing Wang, Yulin Li, Shengpeng Wang, Ling Tao, Wenjun Yan

**Affiliations:** ^1^ Department of Cardiology Xijing Hospital Fourth Military Medical University Xi'an 710032 China; ^2^ School of Public Management Northwest University Xi'an 710127 China; ^3^ Key Laboratory of Aerospace Medicine of the Ministry of Education School of Aerospace Medicine Fourth Military Medical University Xi'an 710032 China; ^4^ Department of Biomedical Engineering University of Alabama at Birmingham Birmingham AL 35294 United States of America; ^5^ Beijing Anzhen Hospital of Capital Medical University and Beijing Institute of Heart Lung and Blood Vessel Diseases Beijing China; ^6^ Department of Cardiovascular Medicine The First Affiliated Hospital of Xi'an Jiaotong University Xi'an 710069 China; ^7^ Department of Toxicology The Ministry of Education Key Lab of Hazard Assessment and Control in Special Operational Environment Shanxi Key Lab of Free Radical Biology and Medicine School of Public Health Fourth Military Medical University Xi'an 710032 China

**Keywords:** macrophages, myocardial infarction, Piezo1, SLC15A3, SLC7A11

## Abstract

The regulation of macrophage function, particularly that of monocyte‐derived macrophages (MoMs), by mechanical forces during myocardial infarction (MI) remains poorly understood. Consistently upregulated Piezo1 expression in cardiac macrophages and MoMs post‐MI is found. Elevated Piezo1 expression in MoMs directly contributes to increased Piezo1 levels in cardiac macrophages. Myeloid cell‐specific Piezo1‐deficient mice (Piezo1^Lyz2^) exhibit significant improvements in ventricular function/remodeling after MI, accompanied by decreased apoptotic cardiomyocytes and decreased inflammation, increased numbers of macrophages, and increased border zone efferocytosis. In vitro, Piezo1 activation by Yoda1 increased oxygen‐glucose deprivation (OGD)‐induced ferroptosis and impaired MoM efferocytosis. Conversely, Piezo1 deficiency in MoMs decreases ferroptosis and increases efferocytosis. SLC7A11 is shown to mediate Piezo1‐induced defective efferocytosis in MoMs. Piezo1 activation aggravated OGD‐induced macrophage ferroptosis via Ca^2+^ influx followed by SLC15A3 upregulation. Piezo1 upregulated SLC7A11 in macrophages via a Ca^2+^/ATF4‐dependent pathway. MoM‐specific SLC7A11 knockdown significantly increases efferocytosis, reduces cardiomyocyte apoptosis and inflammation, and ameliorates post‐MI left ventricular remodeling and function. In conclusion, early Piezo1 activation in MoMs is identified during MI, which governs the fate and function of recruited macrophages. These data establish an ischemic heart–bone marrow functional network and provide a novel therapeutic strategy in which MoM Piezo1 is targeted for post–MI heart repair.

## Graphical abstract

This study reports early Piezo1 activation in bone marrow monocyte‐derived macrophages (MoMs) during myocardial infarction (MI), which governs the fate and function of recruited macrophages. The recruited MoMs with increased Piezo1 expression exhibit increased ferroptosis and defective efferocytosis, delayed clearance of dead cells and resolution of inflammation, leading to the aggravation of post‐MI cardiac remodeling and heart failure.

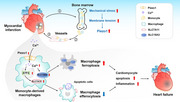

## 1 Introduction

Myocardial infarction (MI), which is pathologically defined as myocardial cell death due to substantial and sustained coronary ischemia, is a highly prevalent disease and a common cause of morbidity and mortality.^[^ ^1^, ^2^ ^]^ Adverse left ventricular remodeling following the major irreversible loss of cardiomyocytes after MI leads to heart failure.^[^ ^3^ ^]^ Multiple cardiomyocyte death programs occur in and around the infarct area due to a reduced O_2_ supply and energy starvation after MI.^[^ ^4^ ^]^ The integrity of the plasma membrane of dying cardiomyocytes is lost, and these cells release their contents, such as mitochondrial DNA, ATP, and double‐stranded RNA, into the surrounding milieu.^[^ ^5^ ^]^ These intracellular components act as danger signals that can be sensed by immune cells and subsequently exacerbate the inflammatory state,^[^ ^6^ ^]^ resulting in further cell death and worsening cardiac function. The effective removal of cellular corpses by phagocytes, also known as efferocytosis, is indispensable for inflammation resolution and wound healing.^[^ ^7^, ^8^ ^]^ Thus, strategies to effectively clear dying cardiomyocytes may provide potential opportunities to retard the progression of post‐MI heart failure.

As critical professional phagocytes, macrophages are indispensable for post‐MI cardiac repair through effective efferocytosis.^[^ ^9^ ^]^ Normally, cardiac macrophages are composed of resident macrophages lacking C‐C Chemokine Receptor 2 (CCR2) and recruited CCR2^+^ cells from circulating monocytes, which are termed monocyte‐derived macrophages (MoMs).^[^ ^10^ ^]^ However, acute MI alters the supply chain of macrophages from tissue‐resident macrophages to MoMs. Within hours after the onset of MI, many resident macrophages die locally.^[^ ^11^ ^]^ Recruited macrophages derived from bone marrow and peripheral blood monocytes populate the ischemic myocardium and function as the main phagocytes.^[^ ^12^ ^]^

MI strongly affects the “seed” or microenvironment of macrophages in the bone marrow. Crosstalk between the ischemic heart and bone marrow extensively affects the function of macrophages. MI changes the bone marrow vascular anatomy and promotes bone marrow angiogenesis and myeloid cell expansion beginning on Day 2.^[^ ^13^ ^]^ In addition, MI induces changes in bone marrow progenitors that lead to myelopoiesis and increased proinflammatory skewing of MoMs.^[^ ^14^ ^]^ Emerging evidences suggest that the phagocytic potential of macrophages is susceptible to the physical properties of the microenvironment, such as substrate stiffness,^[^ ^15^, ^16^ ^]^ indicating a relationship between mechanobiology and macrophage function. However, alterations in mechanosensing pathways in macrophages and their roles in post‐MI remodeling are poorly characterized.

In the present study, we employed high‐throughput data to investigate the expression of mechanosensitive ion channels in cardiac macrophages during MI. We determined the source of cardiac macrophages with high Piezo1 expression, explored the consequences of Piezo1 deficiency on macrophage fate and function in post‐MI hearts, and defined the underlying mechanism(s).

## 2 Results

### 2.1 Piezo1 Expression was Elevated in Cardiac Macrophages After MI

**Figure 1:**
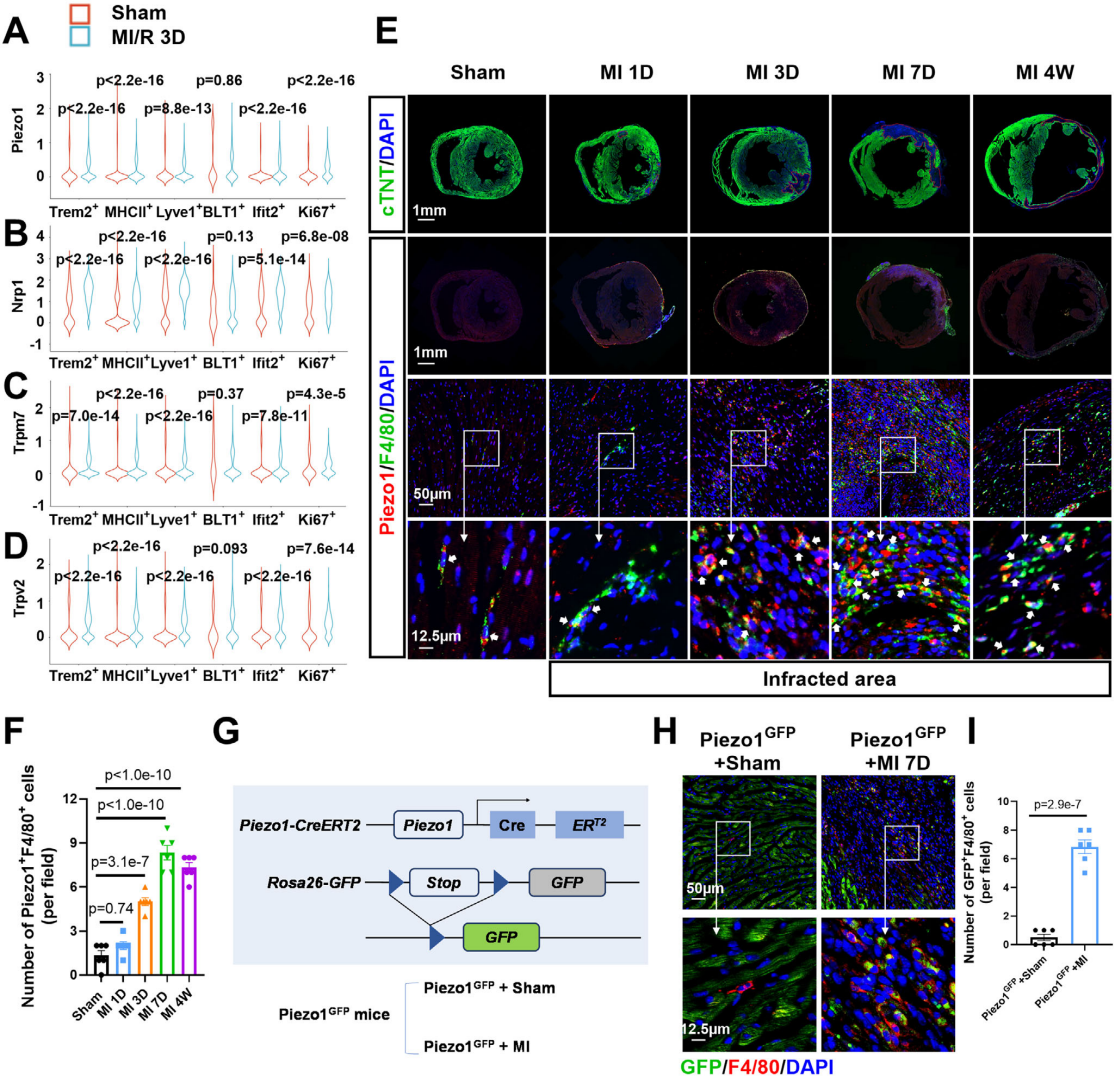
Piezo1 expression in cardiac macrophages increased after MI. A–D) scRNA‐seq analysis of the levels of four mechanosensitive molecules in macrophages from the heart tissues of sham and MI/R‐operated mice (*n* = 22903 cells for sham, *n* = 5042 cells for MI/R 3D). E) Representative images of immunostaining for cardiac troponin T (cTNT), Piezo1, F4/80 and nuclei (DAPI) in murine hearts subjected to the sham or MI procedure. The images of the MI groups were selected from the infarct area. F) Quantification of Piezo1^+^F4/80^+^ cells in (E) (*n* = 6 mice per group). G) Schematic diagram of the experimental procedure performed on Piezo1^GFP^ mice. H) Representative images of F4/80 (red), GFP and nuclei (DAPI, blue) in Piezo1^GFP^ murine hearts. I) Quantification of GFP^+^F4/80^+^ cells in (H) (*n* = 6 mice per group). The data in (A–D) were analyzed using an unpaired 2‐tailed Mann‒Whitney U test. The data in F were analyzed using one‐way ANOVA, followed by the Bonferroni post hoc correction. The data in (I) were analyzed using unpaired Student's *t* test.

Mechanical forces (e.g., shear stress, pressure, stretching, and matrix stiffness) affect tissue and cellular functions in physiological and pathological microenvironments. However, a direct link between mechanical stress and macrophage function in the infarcted heart is still lacking. We previously reported single‐cell RNA sequencing (scRNA‐seq) of macrophages in ischemic heart tissues from mice.^[^ ^17^ ^]^ We reanalyzed the scRNA‐seq, in which the macrophages were classified into six populations by hierarchical clustering analysis (Trem2^+^ macrophages, MHCII^+^ macrophages, Lyve1^+^ macrophages, BLT1^+^ macrophages, Ifit2^+^ macrophages, and Ki67^+^ macrophages). We focused on 10 mechanosensitive molecules (Piezo1, Piezo2, Hsf1, Nrp1, Tmem63a, Tmem63b, Hvcn1, Trpm7, Trpv2, and Trpv4) in these six macrophage populations. Compared with those of the sham hearts, 5 clusters (Trem2^+^, MHCII^+^, Lyve1^+^, Ifit2^+^, and Ki67^+^) of macrophages presented significantly increased mRNA expression of Piezo1, Nrp1, Trpm7, Trpv2, and Hvcn1 3 days after myocardial ischemia/reperfusion (MI/R) (**Figure** **1**A–D; Figure S1A–F, Supporting Information).

Considering that Piezo1 is the most highly expressed mechanosensitive ion channel in macrophages,^[^ ^18^, ^19^ ^]^ we focused on Piezo1 in cardiac macrophages. We performed double immunostaining for Piezo1 and F4/80 in heart sections and found that Piezo1^+^F4/80^+^ cells are mainly located in the infarct and border areas (Figure 1E,F; Figure S1G–I, Supporting Information), and the number of Piezo1^+^F4/80^+^ cells markedly increased 3 days, 7 days, and 4 weeks after MI (Figure 1E–F). Importantly, flow cytometry analyses of single‐cell nonmyocyte suspensions revealed significantly more macrophages and increased Piezo1 expression in macrophages in the ischemic myocardia than in those in the control myocardia (Figure S1J–L, Supporting Information). More than 70% of cardiac macrophages presented high Piezo1 expression 7 days after MI (Figure S1L, Supporting Information).

We constructed Piezo1^GFP^ mice by crossing a recently developed Piezo1‐CreER knock‐in mouse line with the Rosa26 GFP reporter line to better study Piezo1^+^ macrophages in post‐MI heart tissue.^[^ ^20^ ^]^ After tamoxifen induction, the Piezo1^GFP^ mice were subjected to sham and MI operations (Figure 1G). A significant increase in the number of GFP^+^F4/80^+^ cells was observed in the infarcted heart 7 days after MI (Figure 1H,I). Taken together, these results demonstrated for the first time an increased number of macrophages with high Piezo1 expression in post‐MI heart tissues.

### 2.2 Upregulation of Piezo1 in MoMs Contributed to Increased Piezo1 Expression in Cardiac Macrophages Following MI

**Figure 2:**
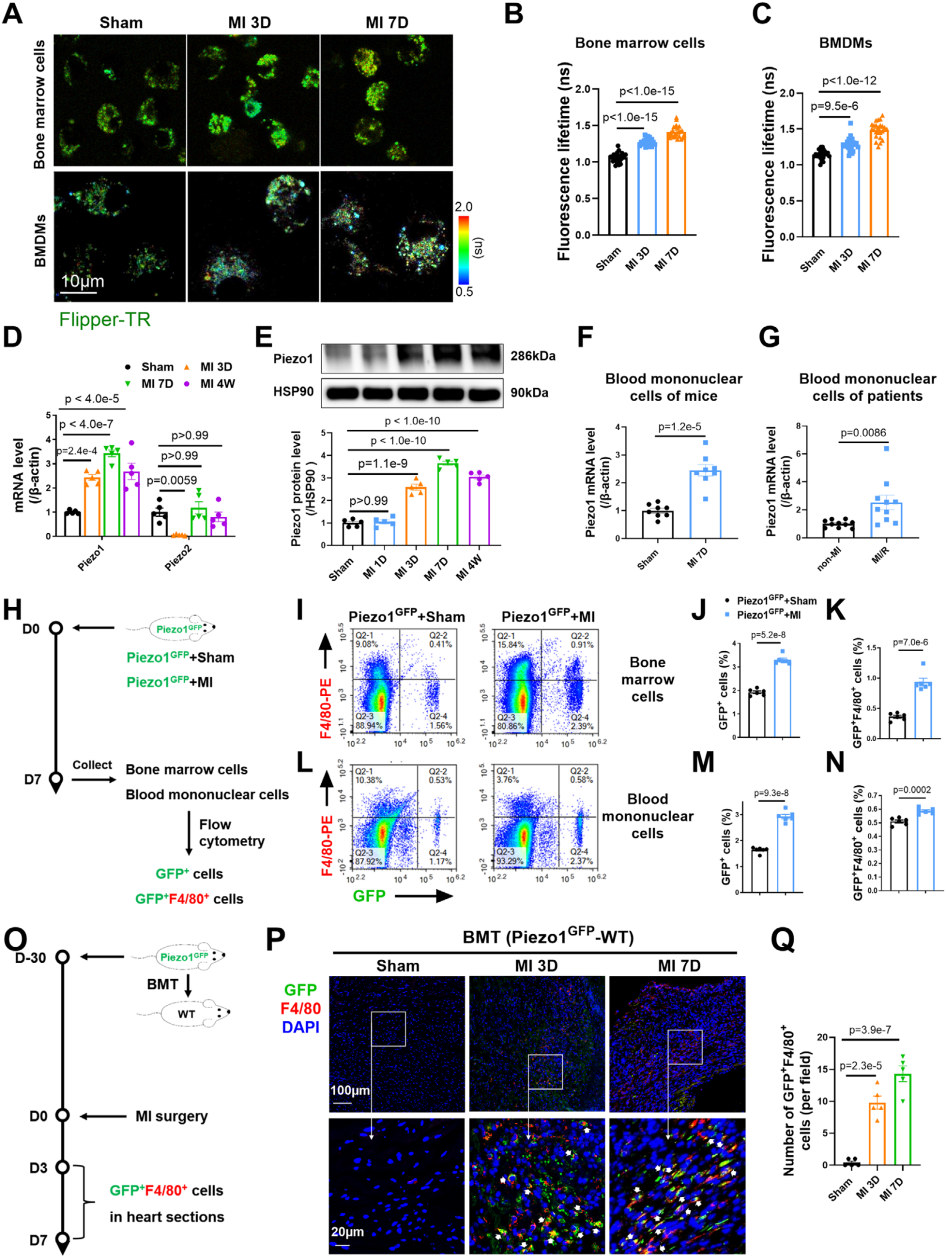
Upregulation of Piezo1 in monocyte‐derived macrophages contributed to increased Piezo1 expression in cardiac macrophages following MI. A) Representative fluorescence lifetime images of freshly isolated bone marrow cells and cultured BMDMs from sham and MI‐operated mice. B,C) The mean lifetime of Flipper‐TR (*n* = 6 mice per group, 4 measurements per animal). D) Quantitative PCR analysis of Piezo1 and Piezo2 expression in BMDMs (*n* = 5 mice per group). E) Representative Western blots and quantification of Piezo1 protein levels in BMDMs (*n* = 5 mice per group). F) Expression of Piezo1 mRNA in blood mononuclear cells (*n* = 8 mice per group). G) Piezo1 mRNA expression in whole blood from patients with acute MI and control patients (*n* = 10 patients per group). H) Schematic diagram showing the experimental design. I–N) Representative fluorescence‐activated cell sorting (FACS) plot of the GFP^+^ and F4/80^+^ subsets and statistics of their frequencies in BMDMs I–K) and blood mononuclear cells L–N) from Piezo1^GFP^ mice (*n* = 6 mice per group). O) Schematic diagram showing the strategy for the bone marrow transplantation (BMT) experiment. P,Q) Representative images and statistics of GFP^+^F4/80^+^ cells in heart tissues from mice that underwent the Sham or MI procedure after BMT (*n* = 5 mice per group). The data in (F,G,J,K,M and N) were analyzed using unpaired Student's *t* test. Other variables were analyzed using one‐way ANOVA, followed by the Bonferroni post hoc correction.

Since cardiac macrophages in the ischemic heart are derived mainly from the bone marrow, we investigated whether Piezo1 is upregulated in MoMs before they reach the infarcted heart. Piezo channels (including Piezo1 and Piezo2) act as mechanosensors, sensing tension in the plasma membrane.^[^ ^21^ ^]^ We performed Flipper‐TR staining to determine the membrane tension of both freshly isolated bone marrow cells and cultured bone marrow‐derived macrophages (BMDMs) from the mice in the sham and MI groups. The membrane tension of the bone marrow cells from the MI group on Days 3 and 7 was significantly greater than that of the bone marrow cells from the sham group (**Figure** **2**A,B). The BMDMs isolated from MI mice also presented increased membrane tension (Figure 2A,C). Consistent with these findings, BMDMs from MI model mice presented significantly increased levels of Piezo1, but not Piezo2, on Day 3 and Day 7 after MI (Figure 2D).

Interestingly, Piezo1 mRNA and protein expression were significantly greater in BMDMs beginning as early as 3 days after MI than in those from sham mice (Figure 2D,E). We detected robust increases in the numbers of Ki67^+^ cells and Piezo1^+^ cells in the bone marrow of Piezo1^GFP^ mice that underwent MI (Figure S2A,B, Supporting Information). Moreover, when BMDMs were subjected to a 0.5 MPa mechanical load, the mRNA and protein expression of Piezo1 was significantly increased (Figure S2C–E, Supporting Information). Therefore, the increased Piezo1 expression in MoMs was probably due to increased mechanical stress in the bone marrow after MI.

Compared with those of the sham controls, the blood mononuclear cells of the MI model mice also presented significantly increased Piezo1 mRNA expression (Figure 2F). We also collected blood mononuclear cells from 10 patients with stable coronary artery disease (non‐MI participants) and 10 patients who successfully underwent percutaneous coronary intervention (reperfusion therapy) after acute MI (MI/R patients, Table S1, Supporting Information). Piezo1 mRNA expression in blood mononuclear cells was significantly greater in the MI/R patients than in the non‐MI participants (Figure 2G).

We then performed flow cytometry to identify Piezo1^+^ macrophages in the bone marrow and blood of Piezo1^GFP^ mice. Compared with those in Piezo1^GFP^+sham mice, significantly more GFP^+^ and GFP^+^F4/80^+^ cells were found in the bone marrow and blood of Piezo1^GFP^+MI mice (Figure 2H–N). We next constructed a mouse model with GFP‐labeled Piezo1 specifically in bone marrow cells by performing bone marrow transplantation from Piezo1^GFP^ mice to wild type (WT) mice. We subjected the transplanted animals to MI surgery (Figure 2O). Immunostaining revealed a significant increase in the number of GFP^+^F4/80^+^ cells in the infarcted heart 3 days and 7 days after MI (Figure 2P,Q). Taken together, these data revealed, for the first time, early increases in membrane tension and Piezo1 expression in MoMs shortly after MI. We provide direct evidence that cardiac macrophages with increased Piezo1 expression were derived, at least in part, from the bone marrow and blood.

### 2.3 Myeloid‐Specific Piezo1 Knockdown Ameliorated Cardiac Dysfunction and Cardiomyocyte Apoptosis After MI

**Figure 3:**
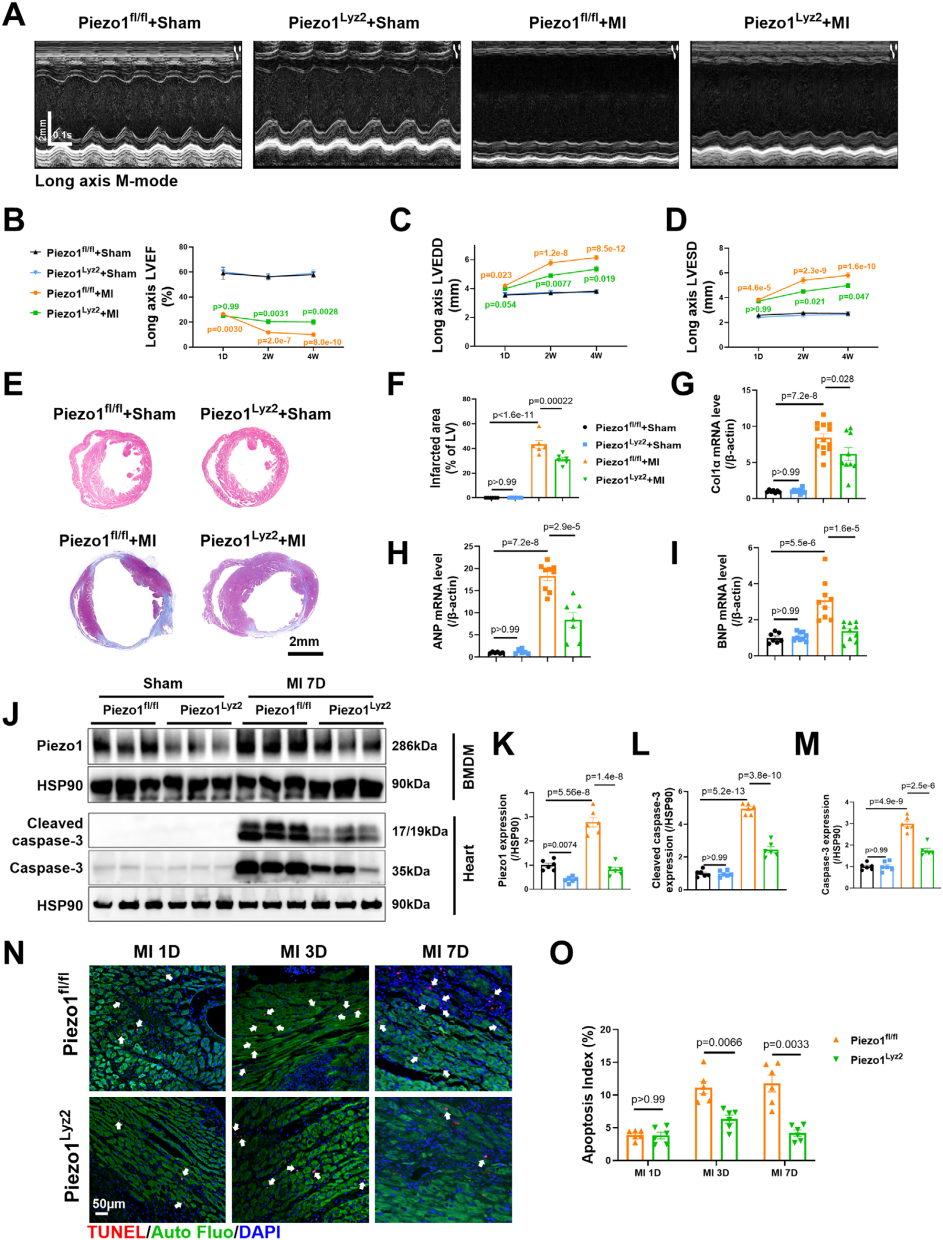
Myeloid deletion of Piezo1 ameliorated cardiac dysfunction and cardiomyocyte apoptosis after MI. A) Representative long‐axis M‐mode echocardiographic images 4 weeks after MI. B–D) The LVEF, LVEDD, and LVESD were evaluated by long‐axis M‐mode echocardiography (*n* = 7, 8, 24, and 25 at 1 D; *n* = 7, 8, 20, and 23 at 2 W; and *n* = 7, 8, 19, and 22 at 4 W). Orange p, compared with the Piezo1^fl/fl^+sham group; green p, compared with the Piezo1^fl/fl^+MI group. E,F) Representative images of Masson's trichrome staining and quantification of the fibrotic area of heart tissue 4 weeks after MI (*n* = 6). G–I) Expression of Col1a, ANP and BNP mRNAs in heart tissues 4 weeks after MI (*n* = 6–13 mice per group). J–M) Representative Western blots and quantification of Piezo1, cleaved caspase‐3 and caspase‐3 protein levels from Piezo1^fl/fl^ and Piezo1^Lyz2^ mice at 7 days after MI (*n* = 6 mice per group). N–O) Representative images and quantification of TUNEL‐positive cardiomyocytes (red, indicated by the white arrows) in the border area at 1 day, 3 days, and 7 days after MI (*n* = 6 mice per group). The data in (B–D) were analyzed using a Mixed‐effects model by Bonferroni's multiple comparisons test. The data in O were analyzed by unpaired Student's *t* test. Other data were analyzed via two‐way ANOVA, followed by the Bonferroni post hoc correction.

Since Piezo1 expression in neutrophils is much lower than that in macrophages,^[^ ^18^ ^]^ we generated Piezo1^Lyz2^ mice by crossing Piezo1^fl/fl^ mice with Lyz2‐Cre mice to explore the function of MoM Piezo1 in regard to MI.^[^ ^19^ ^]^ BMDMs isolated from Piezo1^Lyz2^ mice presented significantly reduced Piezo1 mRNA and protein expression (Figure S3A–C, Supporting Information), while Piezo1 expression level in hearts, livers, lungs, and kidneys remained comparable between the two groups (Figure S3D–G, Supporting Information). Both Piezo1^Lyz2^ and Piezo1^fl/fl^ mice were then subjected to MI or sham surgery. The results of the survival curve demonstrated that there was no statistically significant difference between the two groups after MI operation (Figure S3H, Supporting Information). Cardiac function was determined by consecutive echocardiography in both the long‐ and short‐axis M‐modes 1 day, 2 weeks, and 4 weeks after MI. The echocardiographic parameters of the Piezo1^Lyz2^ mice were comparable to those of the Piezo1^fl/fl^ mice subjected to a sham operation (**Figure** **3**A–D; Figure S3I–L, Supporting Information). Initial cardiac dysfunction was similar between the Piezo1^fl/fl^+MI and Piezo1^Lyz2^+MI groups 1 day after MI, as shown by the comparable left ventricular ejection fraction (LVEF), left ventricular end‐diastolic diameter (LVEDD), and left ventricular end‐systolic diameter (LVESD) values, which were determined by both long‐axis and short‐axis M‐mode echocardiography (Figure 3A–D; Figure S3I–L, Supporting Information). Intriguingly, echocardiographic analysis revealed a significant improvement in heart function 2 and 4 weeks after MI in Piezo1^Lyz2^ mice compared with Piezo1^fl/fl^ mice, as reflected by increased LVEF and decreased LVEDD and LVESD values (Figure 3A–D; Figure S3I–L, Supporting Information). Furthermore, Masson's trichrome staining revealed a significant reduction in the infarct area in Piezo1^Lyz2^ mice 4 weeks after MI (Figure 3E,F). Quantitative PCR analysis of heart tissues verified a significant decrease in the levels of the pathological remodeling markers collagen type I α (Col1α), atrial natriuretic peptide (ANP) and brain natriuretic peptide, (BNP) (Figure 3G–I).

Significantly higher levels of both cleaved and total caspase‐3 proteins were detected in the heart tissues of Piezo1^fl/fl^+MI mice than in those of Piezo1^fl/fl^+sham mice at 1, 3, and 7 days after MI (Figure 3J–M; Figure S3M–R, Supporting Information). Interestingly, the mice in the Piezo1^Lyz2^+MI group presented significantly lower cleaved caspase‐3 and total caspase‐3 protein levels at 3 and 7 days after MI than did the control mice in the Piezo1^fl/fl^+MI group (Figure 3J–M; Figure S3M–R, Supporting Information). We also detected significantly fewer TUNEL‐positive cardiomyocytes in the Piezo1^Lyz2^+MI mice than in the Piezo1^fl/fl^+MI mice at 3 days and 7 days after MI (Figure 3N,O). Taken together, these results indicate that myeloid‐specific Piezo1 knockdown ameliorates adverse ventricular remodeling after MI.

### 2.4 Depletion of Piezo1 in Myeloid Cells Increased the Number and Potentiated the Efferocytotic Activity of Cardiac Macrophages

**Figure 4:**
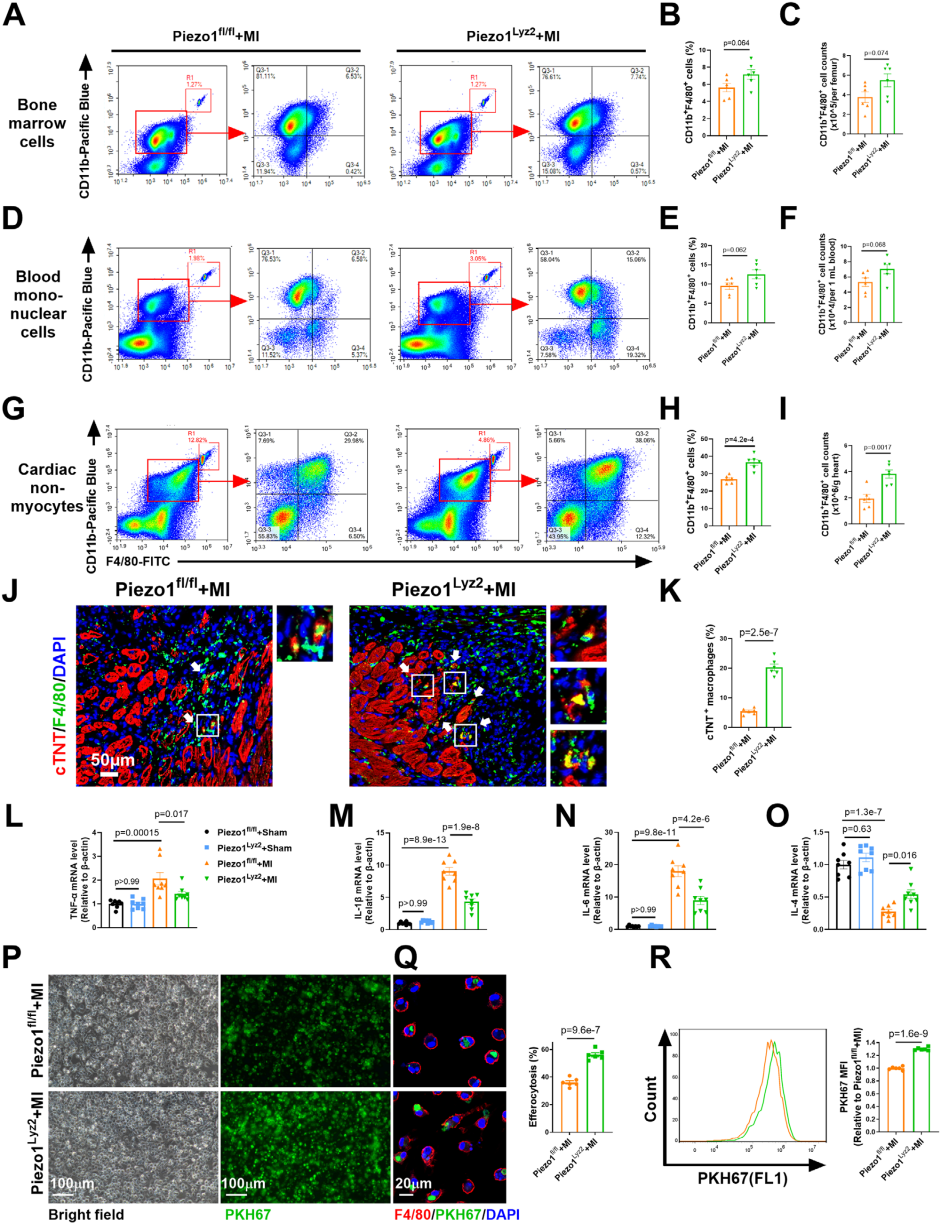
Myeloid deletion of Piezo1 increased the number and potentiated the efferocytotic activity of cardiac macrophages. A–I) Flow cytometry analysis of F4/80^+^CD11b^+^ cells in the bone marrow A–C) and blood D–F) and cardiomyocyte‐depleted cardiac cells G–I) from mice in the Piezo1^fl/fl^+MI and Piezo1^Lyz2^+MI groups on Day 7 (*n* = 6 mice per group). The beads were used to calculate the number of cells. J) Immunofluorescence staining showing the colocalization of F4/80^+^ macrophages (green) with cTNT^+^ cardiomyocytes (red) in the peri‐infarct area 1 week after MI. K) Analysis of the internalization of cardiomyocyte‐derived proteins in macrophages. The macrophages that stained positive for cardiomyocyte cTNT were scored as having internalized cardiomyocyte‐derived proteins (*n* = 6 mice per group). L–O) Expression of the TNF‐α, IL‐1β, IL‐6 and IL‐4 mRNAs in heart tissues 1 week after MI (*n* = 8 mice per group). P) Fluorescence images revealing the uptake of PKH67‐labeled apoptotic Jurkat cells by BMDMs from Piezo1^fl/fl^+MI and Piezo1^Lyz2^+MI mice. Q) Confocal microscopy images and quantification of BMDMs (F4/80, red) that engulfed PKH67‐labeled apoptotic Jurkat cells (green) (*n* = 6 mice per group). R) The uptake of PKH67‐labeled apoptotic Jurkat cells by BMDMs was analyzed via flow cytometry (*n* = 6 mice per group). The data in (L–O) were analyzed by two‐way ANOVA, followed by the Bonferroni post hoc correction. Other variables were analyzed via unpaired Student's *t* test.

Next, we investigated how increased Piezo1 expression in MoMs contributes to post‐MI cardiac dysfunction. We first tested the number of macrophages in the bone marrow, blood, and post‐MI hearts by flow cytometry analysis. There were equal numbers of F4/80^+^CD11b^+^ cells in the bone marrow (**Figure** **4**A–C) and blood (Figure 4D–F) of the Piezo1^fl/fl^+MI and Piezo1^Lyz2^+MI groups. However, a significant increase in F4/80^+^CD11b^+^ cells was observed in the hearts of Piezo1^Lyz2^+MI mice compared with those of Piezo1^fl/fl^+MI mice (Figure 4G–I). Piezo1 reportedly exacerbates cell death.^[^ ^22^, ^23^ ^]^ These data suggest that Piezo1 induces macrophage death in the ischemic heart.

Next, we performed double‐staining of heart sections for markers of cardiomyocytes (cTNT) and macrophages (F4/80) to determine whether Piezo1 in macrophages affects the clearance of apoptotic cardiomyocytes. A limited ratio of cTNT^+^F4/80^+^ dots (yellow) was detected in Piezo1^fl/fl^+MI heart sections, whereas the number of cTNT^+^F4/80^+^ cells was significantly increased in the Piezo1^Lyz2^+MI group (Figure 4J,K), indicating increased efferocytotic activity. Consistent with these findings, depletion of Piezo1 in myeloid cells significantly reversed the changes in the expression of 4 inflammatory cytokines (Tumour necrosis factor alpha (TNF‐α), interleukin‐1 beta (IL‐1β), IL‐6, and IL‐4) in post‐MI heart tissues (Figure 4L–O). Moreover, we cocultured BMDMs with apoptotic Jurkat cells (Figure S4A,B, Supporting Information) to determine the effect of Piezo1 on efferocytosis in vitro. Both immunofluorescence staining and flow cytometry revealed greater uptake of apoptotic cells by Piezo1^Lyz2^+MI macrophages than by Piezo1^fl/fl^+MI macrophages (Figure 4P–R).

To determine whether Piezo1 in MoMs affects the paracrine effects of macrophages on cardiomyocyte apoptosis, we collected conditioned medium (CM) from Piezo1^fl/fl^‐BMDMs and Piezo1^Lyz2^‐BMDMs and then subjected cardiomyocytes to oxygen‐glucose deprivation (OGD) with CM. Compared with fresh medium (FM), both Piezo1^fl/fl^‐BMDM‐CM and Piezo1^Lyz2^‐BMDM‐CM significantly decreased cleaved caspase‐3 protein levels and increased cardiomyocyte viability (Figure S4C–F, Supporting Information). However, no significant difference in the effects of Piezo1^fl/fl^‐BMDM‐CM or Piezo1^Lyz2^‐BMDM‐CM on OGD‐induced cell apoptosis was observed (Figure S4C–F, Supporting Information). Overall, these data suggest that Piezo1 in MoMs contributes to increased cardiomyocyte apoptosis and inflammation by inducing macrophage death and impairing macrophage efferocytosis but not by affecting the paracrine antiapoptotic function of macrophages in MI hearts.

### 2.5 Piezo1 Induced Defective Efferocytosis in MoMs via solute carrier family 7 member 11 (SLC7A11) Upregulation

We utilized a Piezo1‐specific activator, Yoda1, to investigate the impact of Piezo1 on macrophage efferocytosis.^[^ ^24^ ^]^ BMDMs from WT mice were treated with 5 µm Yoda1 or DMSO for 12 h before the efferocytosis assays were performed. We observed significantly decreased uptake of apoptotic Jurkat cells by Yoda1‐primed macrophages compared with that by DMSO‐treated macrophages, as evidenced by both immunostaining and flow cytometry (Figure S5A–D, Supporting Information). Similarly, the decreased efferocytosis mediated by Yoda1 was also corroborated in peritoneal macrophages (PMs) (Figure S5E, Supporting Information).

**Figure 5:**
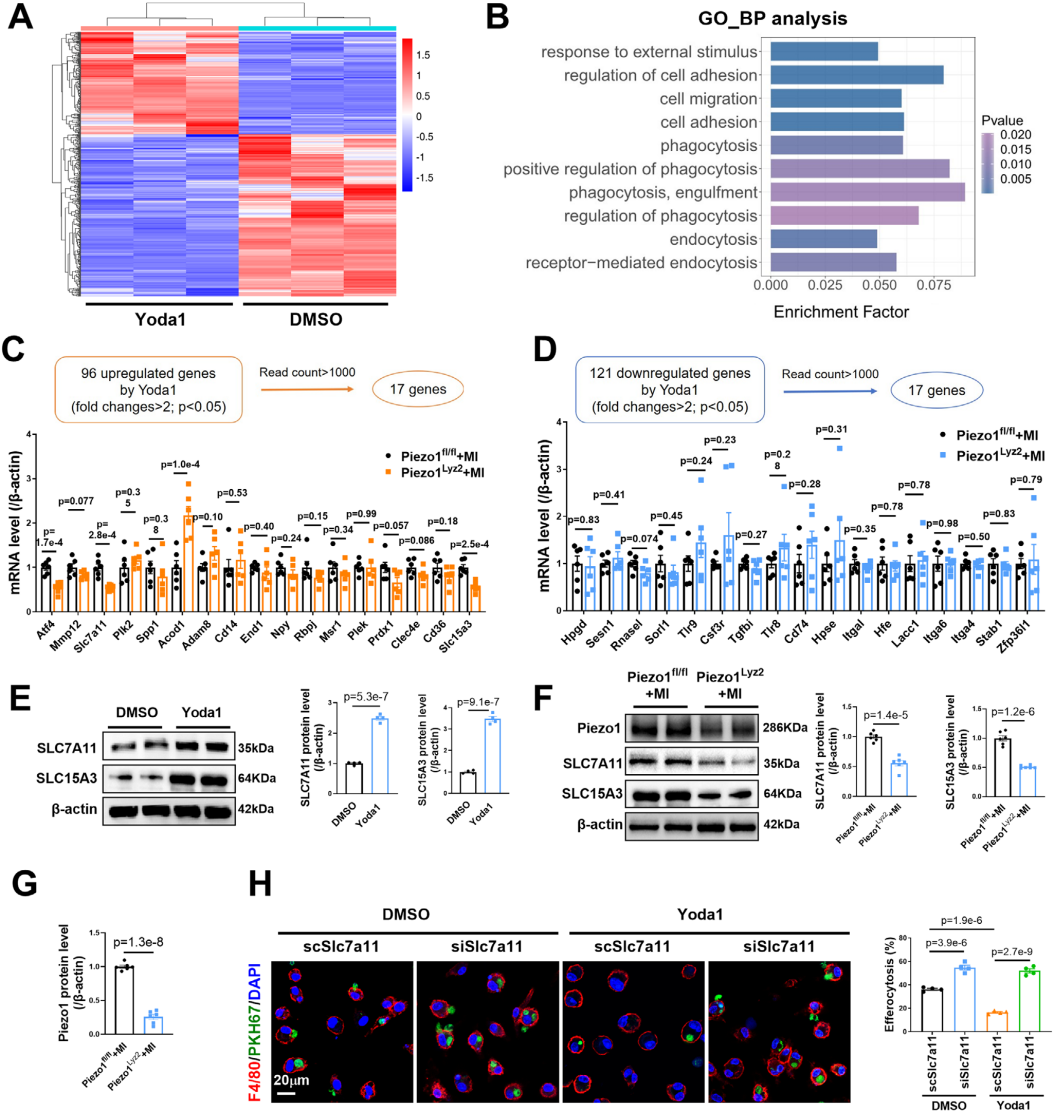
Piezo1 induced defective efferocytosis in MoMs via SLC7A11 upregulation. A) Cluster analysis of the differentially expressed genes (|log_2_Fold Change| > 1, *p* < 0.05) identified by RNA‐seq of BMDMs treated with DMSO or Yoda1 (5 µm) for 12 h) B. GO analysis revealed the enriched biological processes (BPs). C) The 17 genes upregulated according to the GO_BP analysis, including genes with read counts >1000, were analyzed by quantitative PCR in BMDMs from Piezo1^fl/fl^+MI and Piezo1^Lyz2^+MI mice (n = 6 mice per group). D. Quantitative PCR analysis of the 17 genes downregulated according to the GO_BP analysis, including genes with read counts >1000 (*n* = 6 mice per group). E) Representative Western blots and quantification showing SLC7A11 and SLC15A3 protein levels in BMDMs treated with DMSO or Yoda1 (5 µm) for 12 h (*n* = 4). F,G) Representative Western blots and quantification of SLC7A11, SLC15A3 and Piezo1 protein levels in BMDMs from Piezo1^Lyz2^+MI and Piezo1^fl/fl^+MI mice (*n* = 6 mice per group). H) Confocal microscopy images and quantification of BMDMs (F4/80, red) that engulfed PKH67‐labeled apoptotic Jurkat cells (green) (*n* = 4). The data in (C, D, E and F) were analyzed by unpaired Student's *t* test. Other data were analyzed via one‐way ANOVA, followed by the Bonferroni post hoc correction.

To elucidate the molecular mechanisms responsible for Piezo1‐mediated defective efferocytosis, we used a nonbiased RNA‐seq approach to analyze total RNA samples from DMSO‐ and Yoda1‐treated BMDMs. The RNA‐seq analysis revealed 576 genes whose expression differed by more than twofold between the Yoda1 and DMSO groups, among which 223 genes were upregulated and 353 genes were downregulated in the Yoda1 group compared with the DMSO group (**Figure** **5**A). Gene Ontology (GO) analysis revealed that biological processes associated with efferocytosis, which included 96 upregulated genes and 121 downregulated genes, were significantly enriched (Figure 5B). According to their read counts (>1000) in the RNA‐seq data, we focused on 17 upregulated genes and 17 downregulated genes (Figure 5C,D; Table S2, Supporting Information). Since BMDMs from Piezo1^Lyz2^+MI mice presented greater efferocytosis than those from Piezo1^fl/fl^+MI mice, we then screened the above 34 genes in BMDMs from these two groups of mice. The expression of most genes was not significantly different, except for activating transcription factor 4 (Atf4), Slc7a11, Slc15a3, and aconitate decarboxylase 1 (Acod1). Compared with those in Piezo1^fl/fl^+MI BMDMs, Atf4, Slc7a11, and Slc15a3 expression was significantly decreased, whereas Acod1 expression was significantly increased in Piezo1^Lyz2^+MI BMDMs (Figure 5C,D).

We then focused on SLC7A11 and SLC15A3 because they are membrane proteins that are more likely to affect efferocytosis.^[^ ^25^, ^26^ ^]^ The immunoblotting results further confirmed the upregulation of SLC7A11 and SLC15A3 in Yoda1‐treated BMDMs (Figure 5E). In addition, compared with Piezo1^fl/fl^+MI mouse BMDMs, BMDMs isolated from Piezo1^Lyz2^+MI mice presented significantly lower SLC7A11, SLC15A3, and Piezo1 protein levels (Figure 5F,G). BMDMs from WT mice were transfected with small interfering RNAs (siRNAs) against SLC7A11 (siSlc7a11), SLC15A3 (siSlc15a3), or scrambled RNA (scRNA) for 48 h (Figure S5F,G, Supporting Information), followed by the administration of DMSO or Yoda1 to determine the potential contribution of SLC7A11 and SLC15A3 to macrophage efferocytosis. Efferocytosis assays, including immunofluorescence staining and flow cytometry, revealed that SLC7A11‐deficient macrophages effectively increased their efferocytosis capacity (Figure S5H,I, Supporting Information). Confocal images revealed significantly increased engulfment of apoptotic cells by SLC7A11‐deficient macrophages (Figure 5H). More importantly, SLC7A11 knockdown abolished the negative effects of Yoda1 on macrophage efferocytosis (Figure 5H; Figure S5H,I, Supporting Information). However, SLC15A3 knockdown did not significantly alter the efferocytotic capacity of macrophages (Figure S5J,K, Supporting Information). These findings provide solid evidence that Piezo1 activation in macrophages suppresses efferocytosis through the upregulation of SLC7A11 expression.

### 2.6 Piezo1 Exacerbated OGD‐Induced MoM Ferroptosis by Upregulating SLC15A3

Piezo1 has been reported to induce apoptosis and ferroptosis in several cell types,^[^ ^22^, ^23^, ^27^, ^28^ ^]^ but its role in macrophage death is still largely unknown. We subjected BMDMs to OGD, a model used for mimicking the ischemic environment in post‐MI hearts, with or without Yoda1. The viability of the macrophages was reduced by OGD and further decreased by Yoda1 (Figure S6A, Supporting Information). RNA‐seq data of these 4 groups of BMDMs revealed significantly increased ferroptosis score in OGD+Yoda1 group than that in OGD+DMSO group (Figure S6B,C, Supporting Information). Consistently, Yoda1 reduced macrophage apoptosis but triggered ferroptosis in BMDMs subjected to OGD because Yoda1 decreased the protein expression of cleaved caspase‐3 and glutathione peroxidase 4 (GPX4), increased prostaglandin‐endoperoxide synthase 2 (PTGS2) protein expression (Figure S6D–G, Supporting Information), increased the intracellular Fe^2+^ level, as quantified by FeRhoNox‐1 staining (Figure S6H‐6I), and increased the level of reactive oxygen species (ROS), as evaluated by DCFH‐DA staining (Figure S6J,K, Supporting Information). We also measured the level of lipid peroxidation in BMDMs using the C11‐BODIPY^581/591^ probe, which is a hallmark of ferroptosis. OGD significantly increased lipid peroxidation in BMDMs, which was further increased by Yoda1 (Figure S6L, Supporting Information). We then evaluated in vivo macrophage ferroptosis by flow cytometry analysis of cardiomyocyte‐depleted cardiac cells. The level of oxidized C11‐BODIPY in F4/80‐marked macrophages was significantly lower in hearts from Piezo1^Lyz2^+MI mice than in those from Piezo1^fl/fl^+MI mice (Figure S6M, Supporting Information). These results suggest that Piezo1 triggers OGD‐induced MoM ferroptosis.

Importantly, OGD decreased SLC7A11 protein but increased SLC15A3 protein expression, the latter of which was further increased by Yoda1 (Figure S7A–C, Supporting Information). To investigate the role of SLC15A3 in Piezo1‐induced macrophage ferroptosis, we transfected BMDMs with SLC15A3‐targeting siRNA (siSlc15a3). SLC15A3 knockdown largely attenuated Yoda1‐induced macrophage ferroptosis under OGD conditions, as indicated by increased protein expression of GPX4, decreased ROS and lipid peroxidation, and decreased PTGS2 protein expression (Figure S7D–H, Supporting Information). These results indicate that Piezo1 activation plays an important role in MoM ferroptosis occurrence via the upregulation of SLC15A3.

### 2.7 Piezo1 Increased SLC7A11 in a Ca^2+^/ATF4‐Dependent Manner and Increased SLC15A3 in a Ca^2+^‐Dependent Manner in MoMs

**Figure 6:**
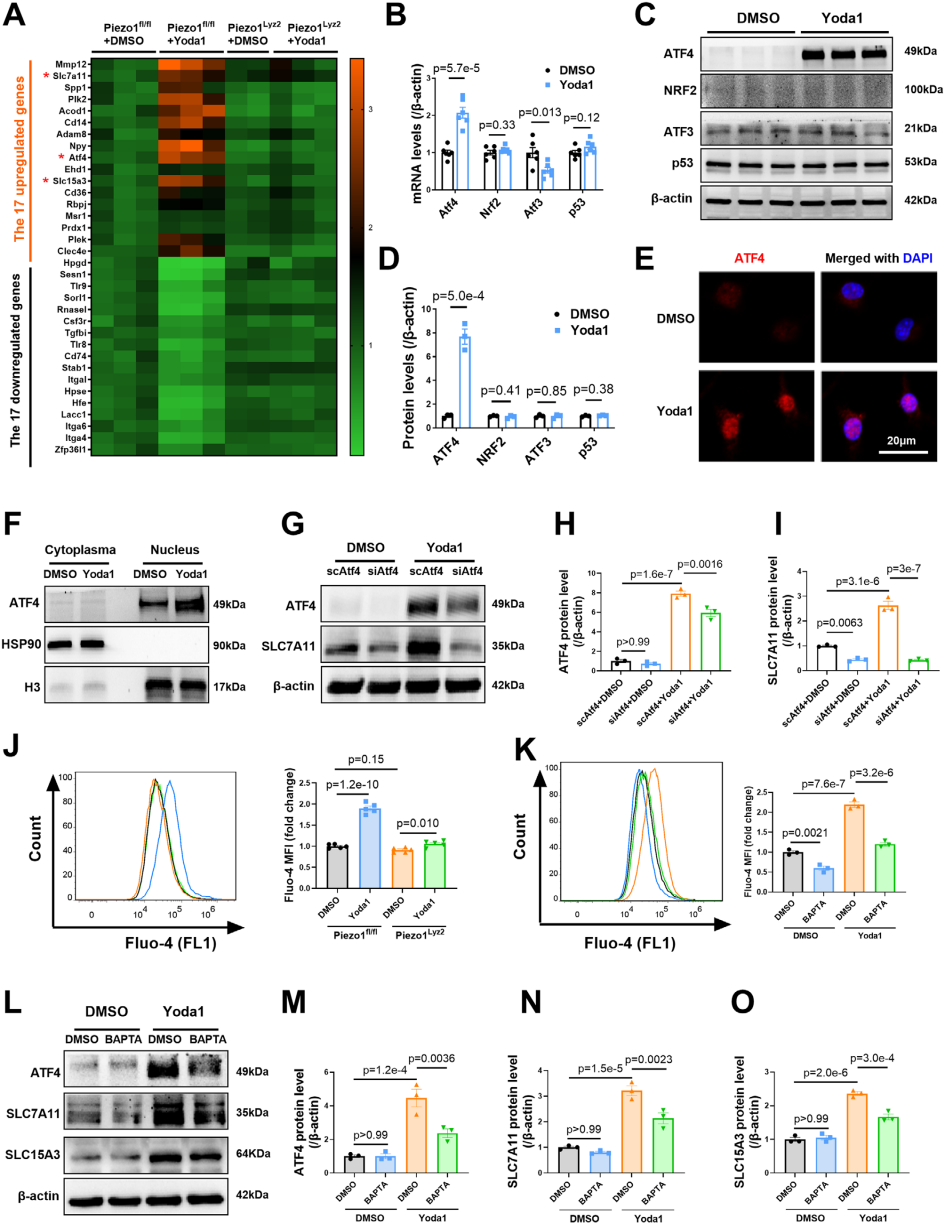
Piezo1 increased SLC7A11 in a Ca^2+^/ATF4‐dependent manner and increased SLC15A3 in a Ca^2+^‐dependent manner in macrophages. A) Heatmap of the genes of interest (the same as the 17 genes that were upregulated and the 17 genes that were downregulated by Yoda1 shown in Figure 5) identified by a new RNA‐seq analysis of Piezo1^fl/fl^ and Piezo1^Lyz2^ BMDMs, which were treated with DMSO or Yoda1 (5 µm) for 12 h. B) Levels of Atf4, Atf3, Nrf2 and P53 mRNAs in BMDMs after treatment with DMSO or Yoda1 (*n* = 6). C,D) Representative Western blots and quantification of the protein levels of ATF4, ATF3, NRF2 and P53 in BMDMs (*n* = 3). E) Representative images of immunofluorescence staining showing the ATF4 level and location in BMDMs treated with DMSO or Yoda1. F) Protein expression of ATF4 in the cytoplasm and nucleus of BMDMs treated with DMSO or Yoda1. G–I) Representative Western blots and quantification of ATF4 and SLC7A11 protein levels in BMDMs after control or Atf4 siRNA transfection and DMSO or Yoda1 treatment (*n* = 3). J) Flow cytometry analysis of intracellular Ca^2+^ levels by Fluo‐4 staining in BMDMs from Piezo1^fl/fl^ and Piezo1^Lyz2^ mice treated with DMSO or Yoda1 for 30 min (*n* = 5 mice per group). K) Fluo‐4 fluorescence signals in BMDMs pretreated with 5 µM BAPTA‐AM for 24 h followed by DMSO or 5 µm Yoda1 for 30 min were analyzed via flow cytometry (*n* = 3). L–O) Representative Western blots and quantification of the protein levels of ATF4, SLC7A11 and SLC15A3 in BMDMs (*n* = 3). The data in (B and D) were analyzed by unpaired Student's *t* test. Other data were analyzed by one‐way ANOVA, followed by the Bonferroni post hoc correction.

Next, we attempted to understand how Piezo1 increases SLC7A11 and SLC15A3 expression in MoMs. We performed RNA‐seq using BMDMs from Piezo1^fl/fl^ and Piezo1^Lyz2^ mice that were treated with DMSO or Yoda1. We reanalyzed the abovementioned 17 Yoda1‐induced upregulated genes and 17 downregulated genes (Figure 5C,D). Yoda1 administration resulted in consistent alterations in these 34 genes in Piezo1^fl/fl^ BMDMs (**Figure** **6**A). More importantly, the Yoda1‐induced alterations in all 34 genes, including Atf4, Slc7a11, and Slc15a3 (Figure 6A), were blocked in Piezo1^Lyz2^ BMDMs. Yoda1‐induced ATF4, SLC7A11, and SLC15A3 upregulation at the mRNA (Figure S8A–C, Supporting Information) and protein (Figure S8D–G, Supporting Information) levels was completely blocked by Piezo1 deficiency in macrophages.

SLC7A11 expression is regulated in a sophisticated manner at the transcriptional level by four transcription factors: the transcriptional activators ATF4 and nuclear factor erythroid 2‐related factor 2 (NRF2), and the transcriptional repressors ATF3 and p53.^[^ ^29^ ^]^ We therefore examined the mRNA and protein levels of these four transcription factors in macrophages after the administration of Yoda1. Yoda1 significantly increased the ATF4 mRNA level and decreased the ATF3 mRNA level but had no significant effect on the mRNA expression of NRF2 or p53 (Figure 6B). Western blotting further confirmed the increase in ATF4 protein expression after Yoda1 treatment (Figure 6C,D). Yoda1 did not significantly alter the protein expression of ATF3, NRF2, or p53 in macrophages (Figure 6C,D).

Next, we performed two experiments to verify the contribution of ATF4 to Yoda1‐induced SLC7A11 transcriptional regulation in macrophages. First, we tested the cytoplasmic and nuclear distributions of ATF4 in BMDMs with or without Yoda1 treatment. Immunofluorescence staining revealed that ATF4 was expressed mainly in the nucleus and was significantly increased by Yoda1 compared with DMSO (Figure 6E). Immunoblotting of ATF4 also revealed increased levels of ATF4 in the nuclei of macrophages (Figure 6F). ATF4 protein expression was nearly undetectable in the cytoplasm (Figure 6E,F). Second, we knocked down ATF4 in BMDMs via siRNA transfection before Yoda1 administration (Figure S8H, Supporting Information). Indeed, ATF4 knockdown significantly reversed the upregulation of SLC7A11 caused by Yoda1 at both the mRNA (Figure S8I, Supporting Information) and protein levels (Figure 6G–I). These data revealed that ATF4 is responsible for the Yoda1‐mediated upregulation of SLC7A11 transcription.

Given that Yoda1‐activated Piezo1 triggers calcium influx,^[^ ^30^ ^]^ we investigated whether the ATF4/SLC7A11 and SLC15A3 pathways are associated with calcium disturbances. Yoda1 induced a significant increase in calcium influx in Piezo1^fl/fl^ BMDMs but not in Piezo1^Lyz2^ BMDMs (Figure 6J). The BMDMs were then treated with the calcium chelator BAPTA‐AM^[^ ^31^ ^]^ before they were exposed to Yoda1. Fluo‐4 staining revealed that BAPTA‐AM caused the elimination of intracellular calcium induced by Yoda1 (Figure 6K; Figure S8J, Supporting Information). Importantly, BAPTA‐AM partially but significantly reduced the mRNA (Figure S8K–M, Supporting Information) and protein expression levels of ATF4, SLC7A11, and SLC15A3 in Yoda1‐treated BMDMs (Figure 6L–O). Taken together, these data demonstrated that Piezo1 activation induces ATF4/SLC7A11 and SLC15A3 upregulation in a Ca^2+^‐dependent manner in MoMs.

### 2.8 MoM‐Specific SLC7A11 Knockdown Restored Macrophage Efferocytosis and Ameliorated Cardiac Dysfunction After MI

**Figure 7:**
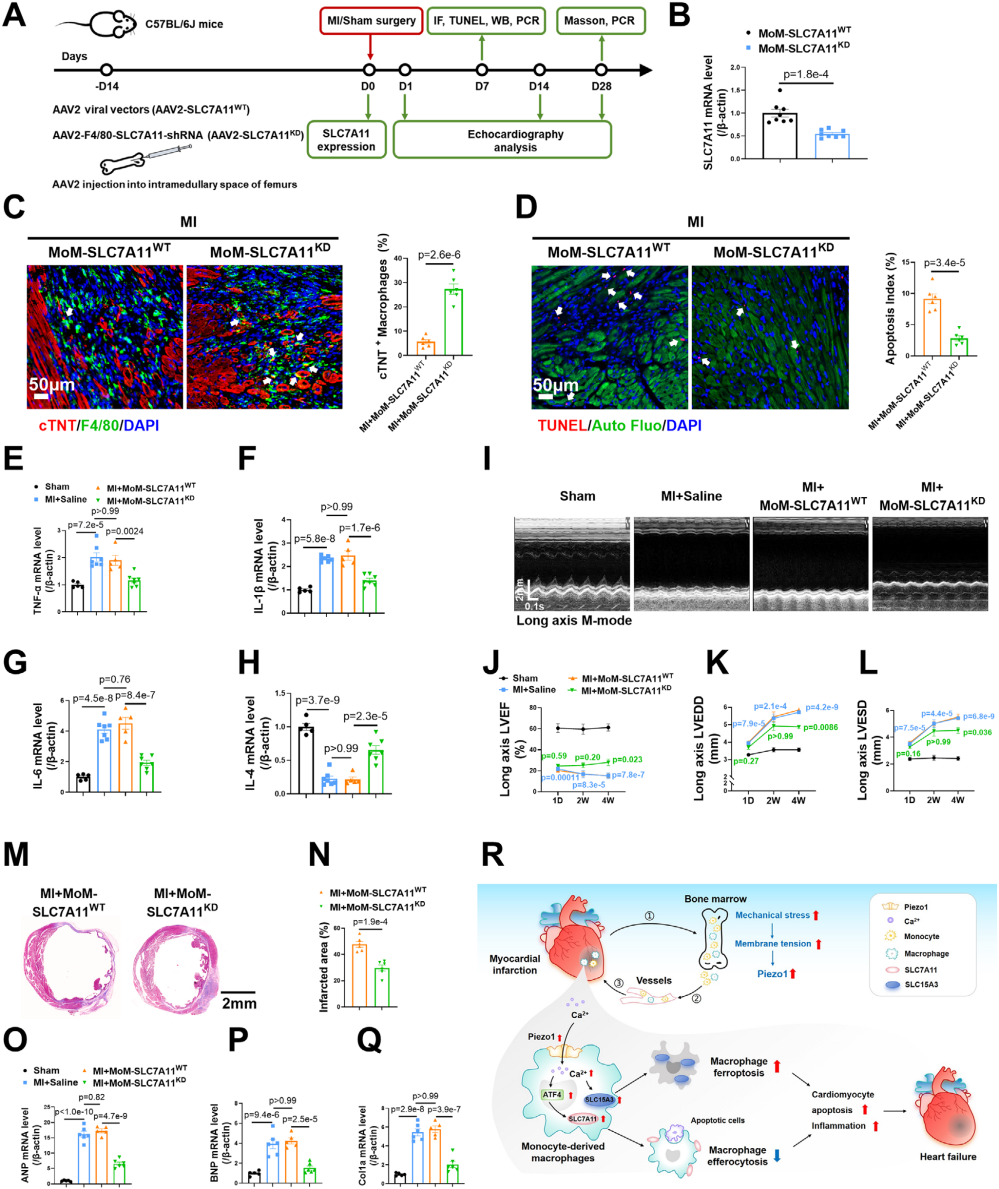
MoM‐specific SLC7A11 knockdown restored macrophage efferocytosis and ameliorated cardiac dysfunction after MI. A) Schematic diagram of the experimental design for MoM‐specific SLC7A11‐knockdown mice. B) Relative SLC7A11 mRNA levels in BMDMs from AAV2‐F4/80‐SLC7A11‐treated and AAV2‐F4/80 empty vector‐treated mice (*n* = 8 mice per group). C) Immunofluorescence staining showing the colocalization of F4/80^+^ macrophages (green) with cTNT^+^ cardiomyocytes (red) in the peri‐infarct area 1 week after MI (*n* = 6 mice per group). D) Representative images of TUNEL‐positive cardiomyocytes (red, white arrows) in the peri‐infarct area 1 week after MI (*n* = 6 mice per group). E–H) Expression of the TNF‐α, IL‐1β, IL‐6 and IL‐4 mRNAs in heart tissues 1 week after MI (*n* = 5–7 mice per group). I) Representative long‐axis M‐mode echocardiographic images 4 weeks after MI. J–L) The LVEF, LVEDD, and LVESD were evaluated via long‐axis M‐mode echocardiography (*n* = 7, 13, 12, and 12 at 1 D; *n* = 7, 9, 8, and 9 at 2 W; and *n* = 7, 9, 8, and 9 at 4 W). Blue p, compared with the sham group; green p, compared with the MI+MoM‐SLC7A11^WT^ group. M,N) Representative images of Masson's trichrome staining and quantification of the fibrotic area of heart tissue 4 weeks after MI (*n* = 6 mice per group). O–Q) The levels of the ANP, BNP and Col1a mRNAs in heart tissues 4 weeks after MI (*n* = 5–6 mice per group). R) Schematic illustration of the Piezo1‐mediated regulation of macrophage ferroptosis and efferocytosis in post‐MI cardiac remodeling. MI results in increased mechanical stress in the bone marrow, which increases membrane tension and Piezo1 expression in bone marrow monocyte‐derived macrophages (MoMs). After being mobilized to the blood and recruited into the ischemic heart, macrophages with high Piezo1 expression exhibit increased Ca^2+^ influx, which increases SLC15A3 expression and nuclear ATF4 expression. ATF4 transcriptionally increases SLC7A11 expression. SLC15A3 exacerbates macrophage ferroptosis, whereas SLC7A11 inhibits macrophage efferocytosis. Dysfunctional MoMs in the heart delay the clearance of dead cells and the resolution of inflammation, leading to the aggravation of post‐MI cardiac remodeling and heart failure. The data in B, C, D and N were analyzed by unpaired Student's *t* test. The data in (J–L) were analyzed using a Mixed‐effects model by Bonferroni's multiple comparisons test. Other variables were analyzed via one‐way ANOVA, followed by the Bonferroni post hoc correction.

Since Piezo1 affects both the fate and function of recruited macrophages, we next determined whether restoring their efferocytotic function would promote post‐MI heart repair. We constructed an AAV2 containing an SLC7A11 short hairpin RNA under the control of a macrophage‐specific F4/80 promoter (AAV2‐F4/80‐SLC7A11‐shRNA). We injected the AAV2‐F4/80‐SLC7A11‐shRNA or AAV2‐F4/80 empty vector into the intramedullary space of the femurs of C57BL/6J mice 2 weeks before performing MI surgery (**Figure** **7**A). SLC7A11 expression was significantly lower in the BMDMs of the AAV2‐F4/80‐SLC7A11‐shRNA‐treated mice (MoM‐SLC7A11^KD^) than in those of the mice treated with the AAV2‐F4/80 empty vector (MoM‐SLC7A11^WT^) 2 weeks after the injection (Figure 7B). One week after MI, we detected significantly greater efferocytotic activity (Figure 7C) and fewer TUNEL‐positive cardiomyocytes (Figure 7D) in heart sections from MI+MoM‐SLC7A11^KD^ mice than in those from control mice. The levels of both the cleaved and total caspase‐3 proteins in heart tissue were significantly lower in the MI+MoM‐SLC7A11^KD^ mice than in the MI + MoM‐SLC7A11^WT^ mice (Figure S9A–C). MoM‐specific SLC7A11 knockdown significantly reversed the changes in the mRNA expression of inflammatory cytokines in 7‐day post‐MI heart tissues (Figure 7E–H). Moreover, compared with MI+MoM‐SLC7A11^WT^ mice, MI+MoM‐SLC7A11^KD^ mice presented significantly improved cardiac function on Week 4 (Figure S9D–G, Supporting Information; Figure 7I–L). Both the infarct area (Figure 7M,N) and the changes in the expression of the pathological remodeling markers ANP, BNP, and Col1α (Figure 7O–Q) were significantly reversed by MoM‐specific SLC7A11 knockdown. Together, these results provide direct in vivo evidence that SLC7A11‐mediated efferocytosis in MoMs attenuates inflammation, adverse ventricular remodeling, and cardiac dysfunction after MI.

## 3 Discussion

One of the increasingly recognized drivers of post‐MI cardiac dysfunction is insufficient efferocytosis, a homeostatic mechanism responsible for the clearance of dead cells and the resolution of inflammation.^[^ ^32^, ^33^, ^34^ ^]^ MI causes the rapid death of cardiac resident macrophages.^[^ ^11^, ^35^ ^]^ However, whether MI directly affects the “seed” of recruited macrophages, i.e., bone marrow MoMs, remains unclear. In the present study, we report several novel findings.

First, we detected significantly increased Piezo1 expression in cardiac macrophages at the early stage after MI. Piezo1 is a mechanosensitive channel discovered in 2010 and represents a breakthrough in the field of mechanotransduction.^[^ ^36^ ^]^ Piezo1 can be activated by various mechanical stimuli that act on cellular membranes, including stretch, poke, shear stress or osmotic swelling.^[^ ^37^ ^]^ In addition, Piezo1 senses the local cellular environment, such as stochastic nanoroughness, confinement or substrate stiffness.^[^ ^38^, ^39^, ^40^ ^]^ Piezo1 upregulation in cardiomyocytes was observed 4 weeks after MI and has been reported to exacerbate lethal ventricular arrhythmogenesis.^[^ ^41^ ^]^ A recent study revealed that MI increases Piezo1 expression in thoracic dorsal root ganglion neurons beginning 3 days after MI, which mediates a neurogenic inflammatory cascade.^[^ ^42^ ^]^ By integrating scRNA‐seq, immunostaining, and data from GFP‐labeled Piezo1 knock‐in mice, we demonstrated, for the first time, significant upregulation of Piezo1 in cardiac macrophages as early as 3 days after MI. These results suggest that Piezo1 activation in cardiac macrophages is an early danger signal for post‐MI hearts.

Second, we revealed that the upregulation of Piezo1 in MoMs contributed to increased Piezo1 expression in cardiac macrophages following MI. Upon observing increased mechanosensitive channels in cardiac macrophages post‐MI, we initially attributed this to myocardial stiffness/mechanical stress. Surprisingly, Piezo1 was also significantly elevated in bone marrow cells, BMDMs, and blood mononuclear cells. The bone marrow microenvironment organizes immune surveillance and responses in other parts of the body and mobilizes appropriate inflammatory and immune cells into the bloodstream. We then constructed a mouse model expressing GFP‐labeled Piezo1 in bone marrow cells and detected significantly increased numbers of Piezo1^+^ macrophages in blood and heart sections after MI. These results demonstrated that the increased number of Piezo1^+^ macrophages in post‐MI heart tissue was directly attributed to the mobilization of MoMs from the bone marrow and blood (Figure 7R).

An important question is how MI increases bone marrow mechanical stress. MI stimulates myeloid cell expansion in the bone marrow.^[^ ^13^, ^43^ ^]^ Ambient mechanical pressures in tissues direct cellular behaviors via sophisticated mechanosensory machinery.^[^ ^44^, ^45^ ^]^ In the present study, Ki67 immunostaining of breastbone sections from Piezo1^GFP^ mice revealed increased Ki67^+^ and Piezo1^+^ cells post‐MI. The spatial proximity of these cells suggested compression‐induced mechanical stress. Furthermore, subjecting BMDMs to 0.5 MPa mechanical load significantly increased Piezo1 mRNA and protein expression. Within the confined space of the bone marrow cavity in MI mice and patients with MI, significantly enhanced myelopoiesis,^[^ ^13^ ^]^ potentially generates mechanical stress on resident bone marrow cells, leading to elevated cell membrane tension. Since direct measurement of bone marrow pressure is technically unfeasible, we used Flipper‐TR staining to demonstrate elevated membrane tension in freshly isolated bone marrow cells from MI‐operated versus sham mice. A very recent study^[^ ^46^ ^]^ reported that MI‐enhanced extramedullary hematopoiesis (increased spleen weight/volume and hematopoietic progenitors) is mediated by type I IFN pathways. Notably, type I IFN responses to cardiac injury originate in the bone marrow.^[^ ^47^ ^]^ Crucially, unlike the expandable spleen, the rigid confinement of the bone marrow cannot accommodate cellular expansion. Therefore, while direct measurement remains challenging, it is scientifically reasonable to propose that post‐MI bone marrow proliferation elevates mechanical stress within the cavity.

Third, we showed that Piezo1 activation in macrophages reduces the number and function of MoMs, which worsens cardiac remodeling after MI. Bone marrow MoMs represent a small population in the healthy heart but are recruited in vast numbers to the injured myocardium after MI. The proper number and function of recruited MoMs are essential for the cardiac healing process.^[^ ^48^ ^]^ Piezo1 has been implicated in several functions and properties of macrophages, such as the polarization response,^[^ ^19^ ^]^ innate immunity,^[^ ^18^ ^]^ and ferroptosis vulnerability.^[^ ^22^, ^27^, ^28^ ^]^ Compared with WT mice, myeloid cell‐specific Piezo1‐deficient mice presented significantly fewer apoptotic cardiomyocytes, reduced fibrosis, and improved cardiac function after MI. Consistent with our findings, two previous studies revealed that myeloid‐specific depletion of Piezo1 protected mice from pulmonary fibrosis and renal fibrosis.^[^ ^18^, ^49^ ^]^ Two mechanisms are involved in the effects of macrophages on cardiomyocyte apoptosis: paracrine function and efferocytosis.^[^ ^50^, ^51^ ^]^ Piezo1 deficiency did not significantly alter the paracrine function of BMDMs upon OGD‐induced cardiomyocyte apoptosis. However, both in vivo and in vitro data indicated that Piezo1 aggravates macrophage ferroptosis and inhibits macrophage efferocytosis.

Previous studies have revealed that Piezo1 deficiency in macrophages restrains macrophage inflammation^[^ ^49^ ^]^ and promotes wound healing responses.^[^ ^19^ ^]^ In our study, myeloid cell‐specific Piezo1‐deficient mice presented significantly decreased levels of proinflammatory cytokines (TNF‐α, IL‐1β, and IL‐6) and increased levels of the anti‐inflammatory cytokine IL‐4. Interestingly, our data revealed that Piezo1‐deficient macrophages have significantly increased efferocytotic activity in the border zone. Since macrophage efferocytosis restrains inflammatory mediators and triggers the release of anti‐inflammatory mediators,^[^ ^52^ ^]^ these results indicate that macrophage Piezo1 activation contributes to cardiac remodeling after MI via aggravation of MoM ferroptosis and inhibition of their efferocytosis.

Fourth, we found that Piezo1 induces macrophage ferroptosis via Ca^2+^/SLC15A3. The role of Piezo1 activation in macrophage ferroptosis has never been reported. Here, we revealed an increased number of F4/80^+^CD11b^+^ cells in the heart, but not in the bone marrow or blood, of Piezo1^Lyz2^+MI mice compared with Piezo1^fl/fl^+MI mice. Consistent with the increased number of F4/80^+^CD11b^+^ cells, there was a significantly reduced number of ferroptotic macrophages, as evidenced by the decreased level of oxidized C11‐BODIPY in F4/80‐marked macrophages in hearts from Piezo1^Lyz2^+MI mice. In the absence of mechanical forces, Piezo1 can be activated by Yoda1, which stabilizes the channel in its open state and slows its inactivation kinetics.^[^ ^53^ ^]^ In vitro experiments also revealed that Yoda1 significantly increases the ferroptosis score and PTGS2 protein expression in macrophages under OGD conditions.

Several recent studies reported that activation of the Piezo1 channel exaggerates ferroptosis in several other cell types in an iron influx, calcium influx, or GPX4‐dependent manner.^[^ ^22^, ^27^, ^28^ ^]^ RNA‐seq screening followed by quantitative PCR/Western blotting demonstrated that Yoda1 significantly induced the expression of ATF4, SLC7A11, and SLC15A3 in macrophages and that these changes were largely abolished by Piezo1 deficiency and by a calcium chelator. SLC7A11 is a well‐known molecule that resists ferroptosis. The oligopeptide histidine transporter SLC15A3, which is expressed mainly on the lysosomal membrane of macrophages, is related to the production of reactive oxygen species.^[^ ^26^ ^]^ OGD decreased SLC7A11 expression in macrophages but was not further altered by Yoda1, which could not explain Piezo1‐induced macrophage ferroptosis. However, we revealed that SLC15A3 knockdown by siRNAs significantly decreased OGD‐induced macrophage ferroptosis. These results demonstrated that Piezo1 induces MoM ferroptosis via a novel Ca^2+^‐mediated SLC15A3 upregulation mechanism.

Finally, we identified a new Ca^2+^/ATF4/SLC7A11‐dependent mechanism underlying Piezo1‐mediated defective efferocytosis in MoMs. A recent study reported the role of Piezo1 in the efferocytosis capacity of macrophages, but did not clarify its underlying mechanisms.^[^ ^16^ ^]^ Using siRNAs against ATF4 and SLC7A11, we revealed that the ATF4/SLC7A11 pathway mediates Piezo1‐induced defects in macrophage efferocytosis. SLC7A11, which functions to exchange intracellular glutamate for extracellular cystine, has been identified as a molecular brake on efferocytosis in dendritic cells.^[^ ^54^ ^]^ However, the role of SLC7A11 in macrophages is poorly understood. In the present study, in vitro experiments revealed that SLC7A11 inhibition by gene knockdown not only increased BMDM efferocytosis at the basal level but also restored BMDM efferocytosis in the presence of Yoda1. More importantly, intramedullary injection of AAV2‐F4/80‐SLC7A11‐shRNA significantly increased cardiac macrophage efferocytosis, reduced inflammation, and improved left ventricular remodeling and function after MI. These results identify macrophage SLC7A11 as a new potential target for the treatment of post‐MI heart failure.

Zhang et al. recently reported that the transcription factor Osr2 integrates biomechanical signaling downstream of Piezo1/Ca^2+^, which leads to CD8^+^ T‐cell exhaustion in tumors.^[^ ^55^ ^]^ In the present study, we found that Piezo1 activation increased the mRNA and protein expression of ATF4. ATF4 is a stress‐induced transcription factor, and its role in macrophage efferocytosis has never been reported. ATF4 transcriptionally upregulates SLC7A11 in several cell lines, such as hepatocytes,^[^ ^56^ ^]^ T cells,^[^ ^57^ ^]^ and renal cell carcinoma.^[^ ^58^ ^]^ Using a calcium chelator, we revealed that Ca^2+^ influx mediates Piezo1‐induced ATF4 upregulation. An immunostaining assay revealed that ATF4 was located mainly in the nucleus, which supports its transcriptional role. After Piezo1 activation, increased nuclear ATF4 promotes SLC7A11 expression in macrophages, which is blocked by ATF4 knockdown. Consistent with our observations, a recent study reported that ATF4 mediates macrophage M1 polarization, leading to an increased inflammatory response.^[^ ^59^ ^]^

In conclusion, our results demonstrate a novel role of macrophage Piezo1 in excessive remodeling after MI by aggravating ferroptosis via Ca^2+^/SLC15A3 and restraining efferocytosis in a Ca^2+^/ATF4/SLC7A11‐dependent manner (Figure 7R). This study may help elucidate the pathological mechanisms of MI from the new viewpoint of heart–bone marrow crosstalk and provide a novel therapeutic measure to treat post‐MI heart failure.

## 4 Experimental Section

### Experimental Animals

This study examined male mice because male animals exhibited less variability in phenotype. All animal procedures were reviewed and approved by the Fourth Military Medical University Animal Experimentation Ethics Committee (the approval number IACUC‐20230250), and were performed in accordance with the National Institutes of Health Guide for the Care and Use of Laboratory Animals. All animals were housed in a pathogen‐free environment and had unrestricted access to food and water. Adult male wild‐type C57BL/6J mice (8–10 weeks) were purchased from the Laboratory Animal Center of the Fourth Military Medical University (Xi'an, China). Lyz2‐Cre mice (Shanghai Model Organisms Center, Inc.) were bred with Piezo1‐floxed mice^[^ ^60^ ^]^ to generate mice with myeloid‐specific deletion of Piezo1 (Piezo1^Lyz2^) and the corresponding Piezo1^fl/fl^ littermate controls. Piezo1‐CreER mouse line was described previously.^[^ ^20^, ^61^ ^]^ The Gt(ROSA)26Sor^2Sho/^J(GFP), which is short for Rosa26 GFP reporter mouse line was from The Jackson Laboratory. GFP‐labeled Piezo1 knock‐in mice (Piezo1^GFP^) were generated by crossing the Piezo1‐CreER knock‐in mouse line with the Rosa26 GFP reporter line. The primers for genotyping of Piezo1^GFP^ mice are: 5′‐ AATAGAAAGTCGCGCTCCCC‐3′, 5′‐CGAGCTTATAAAGGCCCGCA‐3′, and 5′‐ CCACTCCCACTGTCCTTTCC‐3′. Male Piezo1^GFP^ mice aged 8–12 weeks were treated with 5×1 mg/d tamoxifen (HY‐13757, MCE) dissolved in corn oil via intraperitoneal injection, and 7 days later, experiments were started. Euthanasia was performed using carbon dioxide, according to AVMA Guidelines for the Euthanasia of Animals (2020) and approved by local animal welfare committees.

### Clinical Samples

This study protocol was reviewed and approved by the Ethical Review Board of Xijing Hospital and adhered to the provisions of the Declaration of Helsinki. Written informed consent was provided by each participant before enrollment in this study. The male and female patients were from the “TARGET STEMI OCT China Trial” (unique identifier: NCT04150016) performed by our group; please visit the following website for details: https://www.clinicaltrials.gov/ct2/show/NCT04150016.

All subjects were recruited who were diagnosed with acute myocardial infarction (AMI) based on the fourth universal definition of myocardial infarction (2018).^[^ ^1^ ^]^ After primary percutaneous coronary intervention surgery, patients were medically treated. Patients with stable coronary artery disease were invited to be included in the non‐MI group. Fasting peripheral blood samples were collected into tubes containing ethylenediaminetetraacetic acid and prepared for RNA isolation. RNA was purified from whole‐blood samples with the QIAamp RNA Blood Mini Kit (Qiagen) according to the manufacturer's protocol.^[^ ^62^ ^]^

### Single‐Cell RNA Sequencing (scRNA‐seq)

To better understand the alterations of mechanosensitive ion channels in macrophages during the early phase of ischemic heart, scRNA‐seq of heart tissues are performed from sham and MI/R‐operated mice using the 10× Genomics platform as previously reported.^[^ ^17^ ^]^ In brief, left ventricular tissues were obtained 3 days from mice after a sham or MI/R operation. Tissues were minced and dissociated into single‐cell suspensions using the Neonatal Heart Dissociation Kit (Miltenyi Biotec) and the gentle MACS Dissociator. Cells were filtered through a 100 µm strainer, centrifuged, and treated with Debris Removal Solution (Miltenyi Biotec). The resulting suspension was resuspended in Dulbecco's PBS with 0.04% BSA and loaded into the 10× Chromium Controller. Single‐cell RNA‐seq libraries were prepared using the Chromium Single Cell 3′ v2 kit (10× Genomics) and sequenced on the Illumina NovaSeq 6000 platform.

Raw sequencing data were processed with CellRanger to align reads to the mouse reference transcriptome (10 mm) and generate a gene‐cell count matrix. Quality control, normalization, and clustering were performed using Seurat v.2.2.1. Low‐quality cells (fewer than 200 or more than 2500 genes detected, or >30% mitochondrial gene content) and genes expressed in fewer than 5 cells were excluded. Data were normalized and log‐transformed, and 2000 highly variable genes were selected for downstream analysis. Cell types were annotated using canonical marker genes, revealing six distinct macrophage clusters. The differences in gene expression of mechanosensitive ion channels between clusters were calculated using the Wilcoxon rank‐sum test (*p* < 0.05).

### MI Model

The adult C57BL/6J mice (male and female) aged 8–10 weeks were anesthetized with 1%–2% isoflurane, and MI was induced via permanent occlusion of the left anterior descending (LAD) coronary artery as described previously.^[^ ^63^ ^]^ The sham‐operated mice underwent the same surgical procedure without LAD ligation and served as controls. Echocardiographic measurements were performed to assess left ventricular function at days 1, 14, and 28 after the operation (VisualSonics VEVO 2100, Canada). At the corresponding time points, the mice were sacrificed, and the samples were harvested for subsequent experiments.

### Isolation of Cardiomyocyte‐depleted Cardiac Cells

The mice were heparinized (10 IU/g body weight) for 5 min and anesthetized via an intraperitoneal injection of a ketamine/xylazine mixture (100 mg kg^−1^ ketamine plus 10 mg kg^−1^ xylazine). The hearts were removed to prepare for “cardiomyocyte‐depleted” cardiac cell isolation. Single cells were enzymatically isolated from the hearts in 0.1% collagenase type II (20 min at 37 °C) in the Langendorff perfusion system, as described previously.^[^ ^64^, ^65^ ^]^ The digested left ventricle was then snipped, followed by dissociation and resuspension of the cardiac cells in 10% fetal bovine serum. A “cardiomyocyte‐depleted” cardiac cell population was obtained by filtering the cardiac cells through a 70 µm mesh followed by a 40 µm mesh.

### Bone‐Marrow Transplantation

The recipient C57BL/6J mice (8–10 weeks) were administered water supplemented with gentamicin 1 week before irradiation. Irradiation was administered by a small animal instrument with Go^[^ ^46^ ^]^ at a dose of 7.5 Gy. The donor bone marrow cells were isolated from the femurs and tibias of Piezo1^GFP^ mice and then injected into the recipient mice via the angular vein within 4 h after irradiation. The recipient mice were housed for 30 days before myocardial infarction surgery.

### Determination of Cardiac Function

For the analysis of cardiac function in the mice, a transthoracic echocardiography system (VisualSonics, Vevo 770) was used.^[^ ^65^ ^]^ Briefly, after being anesthetized with 1.5% isoflurane, the mice were placed in a supine position on a heating pad. The left ventricular ejection fraction (LVEF), left ventricular end‐diastolic diameter (LVEDD), and left ventricular end‐systolic diameter (LVESD) were measured via both long‐axis and short‐axis M‐mode imaging.

### Establishment of a Mouse Model in Which SLC7A11 was Knocked Down in MoMs

AAV2‐F4/80‐SLC7A11‐shRNA, i.e., an AAV2 viral vector containing a macrophage‐specific F4/80 promoter and SLC7A11 shRNA, was constructed by Likely Biotechnology (Beijing, China). AAV2 containing the F4/80 promoter without the shRNA (AAV2‐F4/80) was constructed as a control viral vector and injected in the same manner. 60 µL of AAV2‐F4/80‐SLC7A11‐shRNA or AAV2‐F4/80 (1 × 10^13^ vector genomes/mL) was injected into the intramedullary space of the femurs of 6‐week‐old male C57BL/6J mice.^[^ ^66^ ^]^ The animals in the control group were injected with saline. Two weeks later, the BMDMs were harvested to determine the knockdown efficacy.

### Isolation and Culture of Macrophages

BMDMs were prepared according to previously described methods.^[^ ^18^ ^]^ Briefly, BMDMs were acquired from tibias and femurs and maintained in DMEM (high glucose, HyClone, South Logan) supplemented with 10% fetal bovine serum, 1% penicillin‒streptomycin and 50 ng mL^−1^ recombinant mouse macrophage colony‐stimulating factor 1 (M‐CSF, Proteintech, Wuhan, China) for 7 days. For these experiments, BMDMs were gently harvested with Enzyme‐free Cell Dissociation Solution (ScienCell, California) and reseeded in new plates for experimentation.

PMs were extracted 3 days after the intraperitoneal injection of a 6% starch‐broth solution, as previously described.^[^ ^67^ ^]^ Briefly, peritoneal lavage fluid was collected in 8 mL of cold phosphate‐buffered saline (PBS), followed by centrifugation to pellet the cells. The cells were plated as needed in complete high‐glucose DMEM. After 3 h, the nonadherent cells were discarded, and fresh medium (FM) was added to cultivate the cells. All the cells were cultured at 37 °C in a humidified 5% CO_2_ and standard oxygen (21%) cell incubator.

### Flow Cytometry Analysis

Bone marrow cells, blood mononuclear cells, “cardiomyocyte‐depleted” cardiac cells, and BMDMs were extracted and washed twice with PBS. For flow cytometry, 2 × 10^6^ cells were incubated with fluorescent dye‐conjugated antibodies in staining buffer (PBS + 0.5% bovine serum albumin + 2 mm EDTA, pH = 7.3) at 4 °C for 30 min. PE anti‐mouse F4/80 antibody (#123110, BioLegend), FITC anti‐mouse F4/80 antibody (#123107, BioLegend), Pacific Blue anti‐mouse/human CD11b antibody (#101224, BioLegend), Pacific Blue anti‐mouse F4/80 antibody (#123124, BioLegend), Precision Count Beads (#424902, BioLegend), and BODIPY 581/591 C11 (2454239, Invitrogen) were used to detect and analyze macrophages. Following the incubation, the cells were washed three times with staining buffer and then centrifuged at 300×g for 5 min. The supernatants were aspirated and discarded. The stained cells were subsequently resuspended in PBS for flow cytometry (BD LSRFortessa). Data were analyzed with Novoexpress software.

### Determination of Membrane Tension

Primary bone marrow cells and BMDMs isolated from mice subjected to the MI or sham procedure were incubated with 1 µm of the membrane tension probe Flipper‐TR (CY‐SC020, Cytoskeleton) for 30 min at 37 °C, as described previously.^[^ ^60^ ^]^ Flipper‐TR, which readily partitions into the plasma membrane and is sensitive to changes in membrane tension, enables noninvasive measurements of membrane tension.^[^ ^68^ ^]^ An increase in the membrane tension prevents rotation of the probe, increasing its fluorescence lifetime. The cells were then washed 3 times with PBS and imaged with a Leica microscope (Stellaris 5Wll). Excitation was performed via a pulsed 488 nm laser (laser kit WLL2+ pulse picker, Leica Microsystems) operating at 80 MHz. For lifetime information, the mean weighted arrival time from the membrane regions was used to report the membrane tension.

### In Vitro Compression of Macrophages

Compression stress was exerted on BMDMs by a pressure incubator according to a previous study.^[^ ^69^ ^]^ The culture system comprised a compression culture chamber, an electrothermostatic water bath, and controllable gas cylinder (Taikang Bio‐Technology, Xi'an, China). To provide compressive stress, the culture chamber was linked with a high‐pressure gas cylinder, which was filled with 76% N_2_, 19% O_2_, and 5% CO_2_. The culture chamber works with compressed gas from the cylinder to the culture dishes, leading to compression of fluid media to the cells under controlled pressure. BMDMs were subjected to controllable compressive stress at 0.25 or 0.5 MPa (excessive mechanical loading) for 24 h. Cells cultured at similar condition without compression loads were used as control (0 MPa).

### Immunofluorescence Staining

Murine hearts and spleens were harvested, embedded in paraffin, and then cut into 5 µm‐thick sections. Cultured macrophages were fixed with 4% paraformaldehyde (PFA, G1101, Servicebio). The heart slices and cells were permeabilized with 0.3% Triton X‐100 and blocked with 5% bovine serum albumin, followed by an incubation with primary antibodies at 4 °C overnight. The signals were visualized with fluorescent dye‐conjugated secondary antibodies (Invitrogen, Carlsbad, CA, USA). The following primary antibodies were utilized for these experiments: F4/80 (GB113373, Servicebio), Piezo1 (28511‐1‐AP, Proteintech), cTnT (GB11364, Servicebio), and ATF4 (#11815, Cell Signaling Technology). Then, 4′‐6‐diamidino‐2‐phenylindole (DAPI) was used to stain the cell nuclei. TdT‐mediated fluorescein‐dUTP nick‐end labeling (TUNEL) assays were performed via a commercially available Roche In Situ Cell Death Detection Kit (Sigma, 11767305001 and 11767291910) according to the manufacturer's instructions. Images were acquired with a fluorescence microscope (Nikon, Tokyo, Japan).

### Fluorescent Ca^2+^ Measurement

Fluo4‐AM (40704ES72, Yeasen, Shanghai) was used to determine the intracellular Ca^2+^ ion concentration.^[^ ^70^ ^]^ BMDMs were plated in 12‐well plates in advance. BMDMs were loaded with 5 mm Fluo4‐AM in Hank's balanced salt solution (HBSS) for 30 min at 37 °C, followed by an incubation with HBSS for 20 min. The Fluo4‐AM fluorescence signal was then detected via fluorescence microscopy and flow cytometry.

### Reactive Oxygen Species Detection

Cellular reactive oxygen species (ROS) level was detected with the oxidation‐sensitive fluorescent dye, 2′,7′‐dichlorofluorescein diacetate (DCFH‐DA) (35845, Sigma‒Aldrich). BMDMs were washed with PBS twice and stained with 10 µm DCFH‐DA in DMEM for 30 min at 37 °C, followed by two washes with PBS. The fluorescent signal was then detected via fluorescence microscopy and flow cytometry.

### Intracellular Fe^2+^ Detection

FeRhoNox‐1 (HY‐D1533, MCE), a commercial fluorescent probe that specifically binds Fe^2+^, was used to determine intracellular Fe^2+^ content. According to the instructions, 10 µm RhoNox‐1 was introduced to BMDMs and incubated for 30 min at 37 °C. The fluorescent signal was further determined by fluorescence microscopy and flow cytometry.

### Lipid Peroxidation Assessment

Lipid peroxidation was assessed by C11‐BODIPY581/591 probe (2454239, Invitrogen) staining. An amount of 5 µm BODIPY‐581/591 C11 was added to the media and incubated for 30 min at 37 °C. The cells were then collected and washed twice with PBS followed by resuspension in PBS. Cell fluorescence was acquired and analyzed by flow cytometry.

### Masson's Trichrome Staining

Four weeks after MI, heart sections were prepared and stained using a Masson's trichrome stain kit (Solarbio, G1340‐100) to assess the area of collagen‐containing fibrous tissue according to the manufacturer's protocol. Masson's trichrome‐stained sections were observed via an optical microscope (Nikon, Japan) and quantified with ImageJ software.

### Efferocytosis Assays

Human Jurkat cells were labeled with the lipophilic dye PKH67 (Sigma‒Aldrich) or CM‐DiI (C7001; Thermo Fisher Scientific). For the induction of apoptosis, Jurkat cells were resuspended in RPMI supplemented with 5% fetal calf serum, irradiated with 150 mJ cm^−2^ ultraviolet C for 15 min, and then incubated at 37 °C with 5% CO_2_ for 2‒3 h.^[^ ^71^ ^]^ After cell counting, the apoptotic Jurkat cells were added to BMDMs at a 5:1 ratio for 45 min.^[^ ^72^ ^]^ The macrophages were vigorously rinsed with PBS to wash out the unbound apoptotic cells and then fixed with 4% paraformaldehyde at room temperature for 15 min. PKH67‐ or CM‐DiI‐positive macrophages were detected via fluorescence microscopy or flow cytometry (Beckman Coulter, Miami, USA).

### siRNA Transfection

siRNAs targeting SLC7A11, SLC15A3, and ATF4 and a negative control (Table S3, Supporting Information) were designed and synthesized by GenePharma (Shanghai, China). siRNAs were transfected into the macrophages with RNAiMAX Transfection Reagent (13778075, Thermo Fisher Scientific) according to the manufacturer's protocol. After 6‒8 h of incubation (37 °C), the siRNA mixture was replaced with complete medium, and the mixture was incubated for 48 h. Afterward, the knockdown efficiency was determined by PCR and Western blotting.

### RNA Extraction and Quantitative Real‐Time Polymerase Chain Reaction (PCR)

Total RNA was extracted from BMDMs and tissue samples and purified with an RNAsimple Total RNA Kit (Qiagen, Hilden, Germany) according to the manufacturer's instructions. One microgram of total RNA was subsequently reverse transcribed into cDNA using a PrimeScript RT reagent Kit with gDNA Eraser (RR047A, TaKaRa, Dalian, China). Gene expression levels were analyzed in triplicate in 10 µL reactions with SYBR Green PCR Master Mix according to the manufacturer's protocol (4472908, Thermo Fisher Scientific). The primers were purchased from TsingKe Biotechnology (Table S4, Supporting Information). Relative mRNA expression levels were normalized to those of β‐actin via the ΔΔCt method.

### Western Blotting

Heart tissues were homogenized, and cultured macrophages were lysed in ice‐cold RIPA lysis buffer containing a proteinase and phosphatase inhibitor cocktail (78438, Thermo Fisher Scientific) and quantified using a bicinchoninic acid (BCA) assay kit (AR1189, Boster). Protein samples were subjected to sodium dodecyl sulfate‒polyacrylamide gel electrophoresis and transferred to polyvinylidene fluoride membranes. The membranes were blocked with 5% fat‐free dried milk, probed with primary and HRP‐conjugated secondary antibodies, and then visualized with an enhanced chemiluminescence (ECL) detection system (Bio‐Rad, CA, USA). The primary antibodies used were specific for Piezo1 (28511‐1‐AP, Proteintech), ATF4 (#11815, Cell Signaling Technology), ATF3 (DF3110, Affinity), Nrf2 (#AF0639, Affinity), P53 (AF0879, Affinity), SLC7A11 (DF12509, Affinity), SLC15A3 (20866‐1‐AP, Proteintech), GPX4 (#DF6701, Affinity), PTGS2 (#AF7003, Affinity), cleaved caspase‐3 (#9661, Cell Signaling Technology), caspase‐3 (#9662, Cell Signaling Technology), HSP90 (#AF6126, Affinity), β‐actin (GB15001‐100, Servicebio), and histone H3 (#9715, Cell Signaling Technology).

### RNA Sequencing (RNA‐seq) Analysis

The differential gene expression analysis was performed via RNA‐seq at Shanghai Personal Biotechnology.^[^ ^73^ ^]^ Total RNA was extracted from the macrophages using TRIzol (Invitrogen, Carlsbad, CA, USA) according to the manufacturer's instructions. The total RNA concentration and purity were determined with a NanoDrop spectrophotometer (Thermo Scientific). Three micrograms of RNA were used as input material to prepare the RNA samples. The sequencing libraries were generated according to the following steps. First, mRNA was purified from total RNA with poly‐T oligo‐attached magnetic beads. Fragmentation was performed using divalent cations in Illumina proprietary fragmentation buffer at an elevated temperature. First‐strand cDNA was synthesized using random oligonucleotides and Super Script II. Second‐strand cDNA synthesis was subsequently performed with DNA polymerase I and RNase H. The remaining overhangs were converted into blunt ends via exonuclease/polymerase activities, and the enzymes were removed. After adenylation of the 3′ ends of the DNA fragments, Illumina PE adapter oligonucleotides were ligated to prepare for hybridization. For the selection of cDNA fragments of the preferred 400–500 bp in length, the library fragments were purified using the AMPure XP system (Beckman Coulter, Beverly, CA, USA). DNA fragments with ligated adaptor molecules on both ends were selectively enriched with the Illumina PCR Primer Cocktail in a 15‐cycle PCR. Products were purified (AMPure XP system) and quantified using the Agilent high‐sensitivity DNA assay on a Bioanalyzer 2100 system (Agilent). The sequencing library was then sequenced on the NovaSeq 6000 platform (Illumina) (Shanghai Personal Biotechnology Co., Ltd.).

### Experimental Drugs

The Piezo1 agonist Yoda1 (HY‐18723) and calcium chelator BAPTA‐AM (HY‐100545) were purchased from MedChemExpress (MCE, Shanghai, China).

### Statistical Analysis

Normality of data distribution was examined using the Shapiro–Wilk normality test. The data are presented as the means ± standard errors of the mean (SEM). The sample size (n) for each experiment was indicated in the corresponding figure legend. Comparisons between two groups were performed using a two‐tailed Student's *t*‐test for normally distributed data. For comparisons involving more than two groups, one‐way ANOVA analysis of variance followed by the Bonferroni post hoc correction was used for normally distributed data. Two‐way ANOVA was performed for datasets involving two independent variables. The echocardiographic data were analyzed by using a Mixed‐effects model by Bonferroni's multiple comparisons test. For data not following a normal distribution, the unpaired 2‐tailed Mann‐Whitney U test (2 groups) was used. All the statistical analyses were performed with GraphPad Prism 9.0 (La Jolla, CA, USA). The results were considered statistically significant when *p* < 0.05.

## Conflict of Interest

The authors declare no conflict of interest.

## Supporting information



Supporting Information

Supporting Information

## Data Availability

All the data needed to evaluate the conclusions in the paper are presented in the paper and/or the Supplementary Materials. The authors declare that all supporting data and materials presented within this article and in the Data Supplement are available from the corresponding author by reasonable request.
