# Supplementary material for: Piezo1 Upregulation in Monocyte‐Derived Macrophages Impairs Post‐Myocardial Infarction Cardiac Repair via Defective Efferocytosis and Enhanced Ferroptosis
Source: Adv Sci (Weinh). 2025 Nov 10;13(5):e10991. doi: 10.1002/advs.202510991 (PMC12850066; doi:10.1002/advs.202510991)
Supplement: Supplementary file 1 — Supporting Information [file ADVS-13-e10991-s001.docx]

**Piezo1 Upregulation in Monocyte-derived Macrophages Impairs Post-myocardial Infarction Cardiac Repair via Defective Efferocytosis and Enhanced Ferroptosis**

**Authors**

Lu Peng^1, #^, Yunlong Xia^1, #^, Huishou Zhao^1, #^, Yongzhen Guo^1,2, #^, Xiaoming Xu^1^, Xue Han^1^, Shiyue Wang^1^, Fengyue Ding^1^, Quanchi Liu^1^, Congye Li^1^, Yuan He^1^, Zhaoyi Luo^1^, Qiaojuan Wang^1^, Xing Zhang^3^, Feng Gao^3^, Yajing Wang^4^, Yulin Li^5^, Shengpeng Wang^6^, Ling Tao^1,^ *, Wenjun Yan^1,7,^ *.

**Affiliations**

1. Department of Cardiology, Xijing Hospital, Fourth Military Medical University, Xi’an 710032, China.

2. School of Public Management, Northwest University, Xi’an 710127, China.

3. Key Laboratory of Aerospace Medicine of the Ministry of Education, School of Aerospace Medicine, Fourth Military Medical University, Xi’an 710032, China.

4. Department of Biomedical Engineering, University of Alabama at Birmingham, AL 35294, United States of America.

5. Beijing Anzhen Hospital of Capital Medical University and Beijing Institute of Heart Lung and Blood Vessel Diseases, Beijing, China.

6. Department of Cardiovascular Medicine, The First Affiliated Hospital of Xi’an Jiaotong University. Xi’an 710069, China.

7. Department of Toxicology, The Ministry of Education Key Lab of Hazard Assessment and Control in Special Operational Environment, Shanxi Key Lab of Free Radical Biology and Medicine, School of Public Health, Fourth Military Medical University, Xi’an 710032, China.

^#^These authors contributed equally to this work.

***Correspondence authors:**

**Wenjun Yan**, M.D., Ph.D.

Associate Professor

Department of Cardiology, Xijing Hospital

The Fourth Military Medical University

127 West Changle Rd, Xi’an, China, 710032

Tel: +86-29-84775183

Fax: +86-29-84771692

E-mail: yanyan517032@163.com (WY);

**Ling Tao**, M.D., Ph.D.

Professor and Chief of the Department of Cardiology,

Xijing Hospital, The Fourth Military Medical University

127 West Changle Rd, Xi’an, China, 710032

Tel: +86-29-84771692, +86-29-84775183

Fax: +86-29-84771692

E-mail: lingtaofmmu@qq.com (LT)

**Supplementary figures and figure legends**


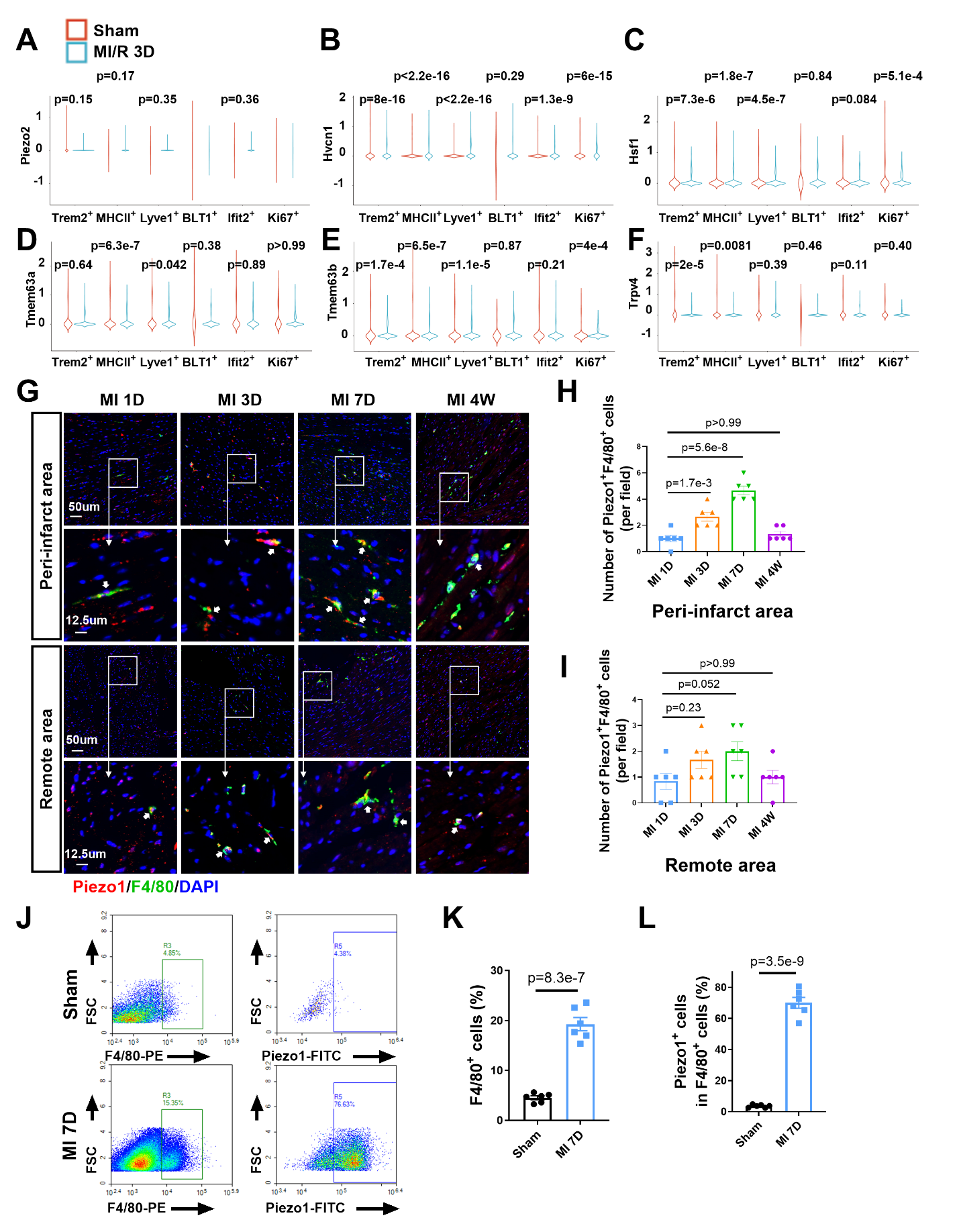


**Figure S1**. **The expression of mechanosensitive molecules in cardiac macrophages from murine hearts**. A-F. Levels of six mechanosensitive molecules in macrophages determined using single-cell RNA sequencing (scRNA-seq) of heart tissues from sham and MI/R-operated mice (n = 22903 cells for the sham group, n = 5042 cells for the MI/R 3D group). G. Representative images of Piezo1 and F4/80 immunostaining in heart tissues. H-I. Quantification of Piezo1^+^F4/80^+^ cells in the border and remote areas (n=6 mice per group). J-L. Flow cytometry analysis of Piezo1^+^ macrophages in single-cell nonmyocyte suspensions from the hearts of mice subjected to the sham or MI procedure for 7 days (n=6 mice per group). The data in A-F were analyzed via an unpaired 2-tailed Mann‒Whitney U test. The data in H-I were analyzed using one-way ANOVA, followed by the Bonferroni post hoc correction. The data in K and L were analyzed via unpaired Student’s *t* test.a


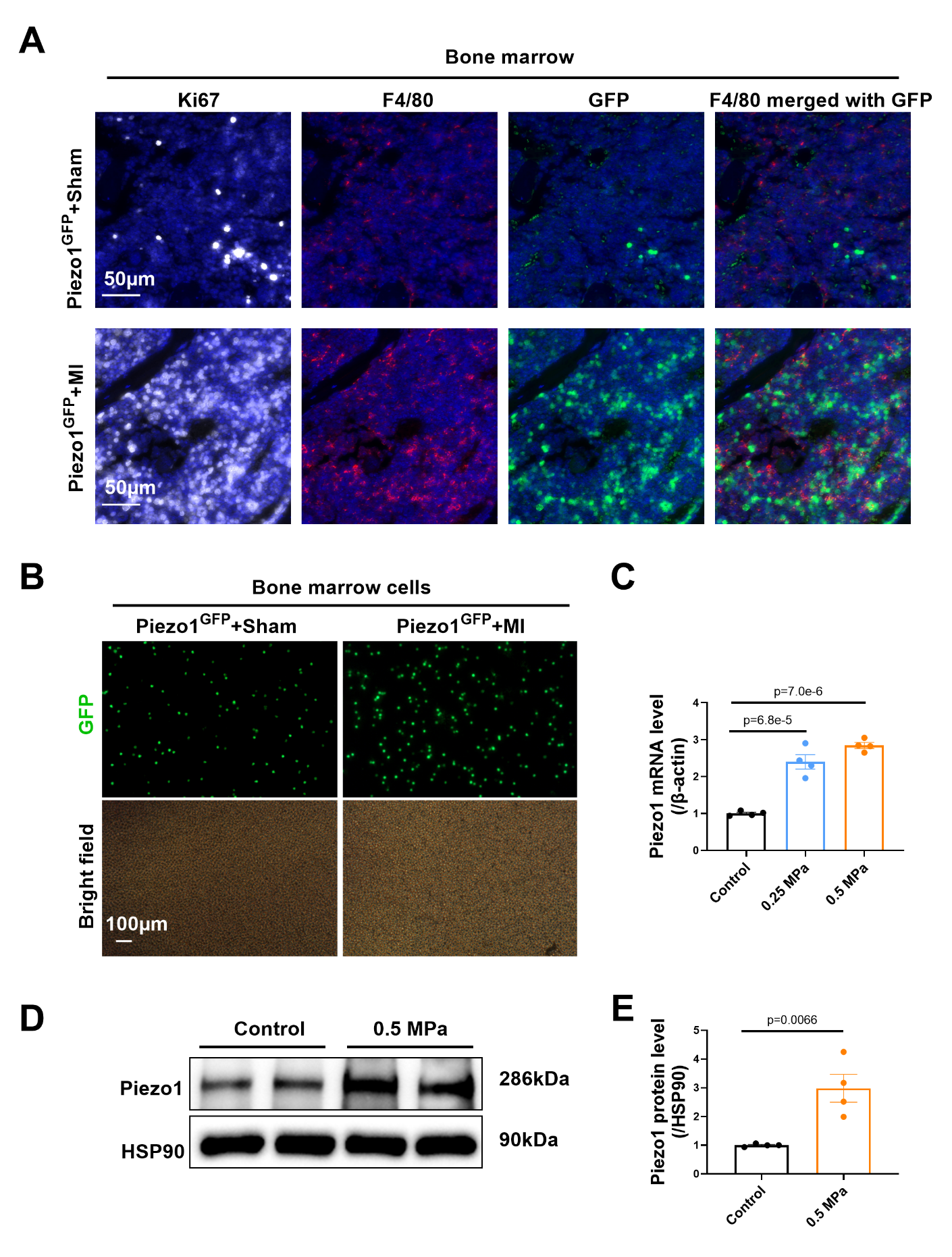


**Figure S2. MI increased Piezo1 expression in bone marrow cells.** A. Representative images of immunostaining for Ki67 (white), F4/80 (red), Piezo1 (GFP, green) and nuclei (DAPI, blue) in the breastbones of Piezo1^GFP^ mice that underwent the sham or MI procedure for 7 days. B. Representative images of GFP staining and bright field images of bone marrow cells from Piezo1^GFP^ mice subjected to the sham or MI procedure for 7 days. C-E. BMDMs were subjected to an in vitro compression model with excessive mechanical load for 24 h. The level of Piezo1 mRNA and protein expression in BMDMs were tested (n = 4). The data in C was analyzed using one-way ANOVA, followed by the Bonferroni post hoc correction. The data in E was analyzed via unpaired Student’s *t* test.


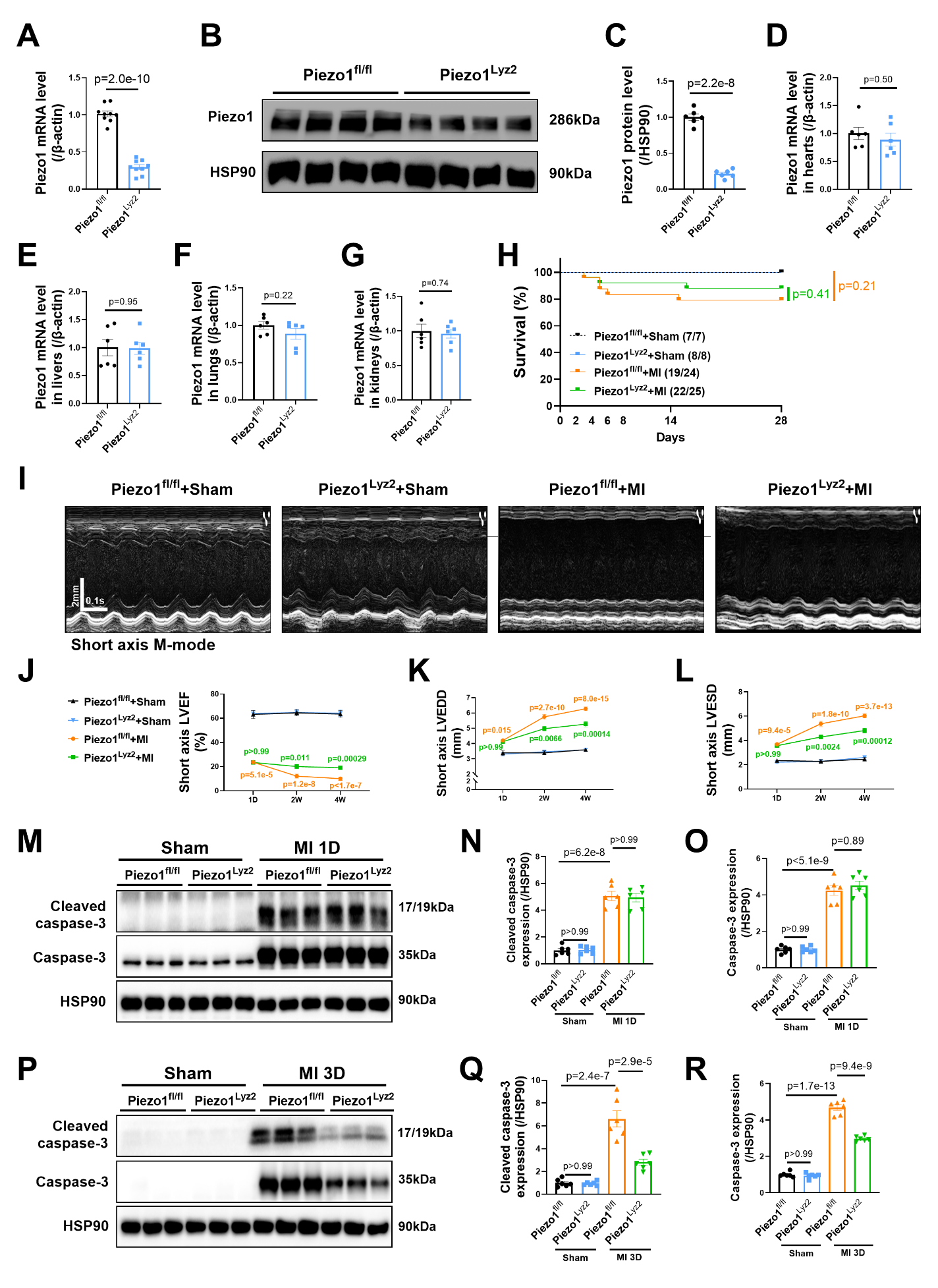


**Figure S3**. **Myeloid deletion of Piezo1 ameliorated cardiac dysfunction after MI.** A. Relative mRNA level of Piezo1 in BMDMs isolated from Piezo1^fl/fl^ and Piezo1^Lyz2^ mice (n=9 mice per group). B-C. Representative Western blot and quantification of Piezo1 protein in BMDMs isolated from Piezo1^fl/fl^ and Piezo1^Lyz2^ mice (n=6 mice per group). D-G. Piezo1 mRNA level in hearts, livers, lungs and kidneys of Piezo1^fl/fl^ and Piezo1^Lyz2^ mice (n=6 mice per group). H. Survival curve. I. Representative short-axis M-mode echocardiographic images at 4 weeks after MI. J-L. The LVEF, LVEDD, and LVESD were evaluated by short-axis M-mode echocardiography (n=7, 8, 24, and 25 at 1 D; n=7, 8, 20, and 23 at 2 W; and n=7, 8, 19, and 22 at 4 W). Orange p compared with the Piezo1^fl/fl^+sham group, and green p compared with the Piezo1^fl/fl^+MI group. M-R. Representative Western blots and quantification of cleaved caspase-3 and caspase-3 protein levels in heart tissues from Piezo1^fl/fl^ and Piezo1^Lyz2^ mice at 3 days (J-L) and 7 days (M-O) after MI (n=6 mice per group). The data in A-G were analyzed by unpaired Student’s *t* test. Survival curve was analyzed by log-rank test. The data in J-L were analyzed using a Mixed-effects model by Bonferroni's multiple comparisons test. Other data were analyzed via two-way ANOVA, followed by the Bonferroni post hoc correction.


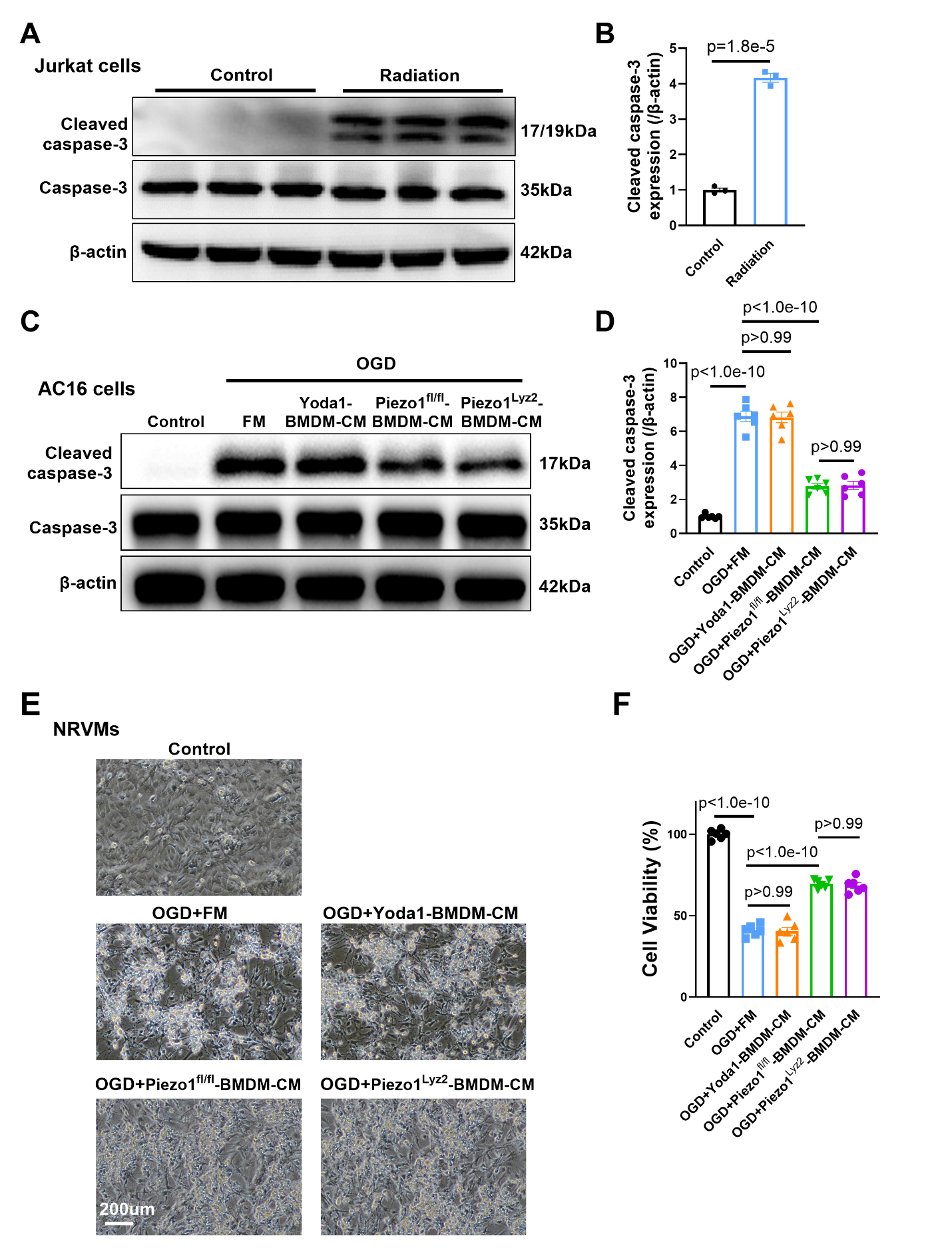


**Figure S4. Piezo1 deficiency did not affect the paracrine anti-apoptotic effects of macrophages on cardiomyocytes.** A-B. Representative Western blots and quantification of cleaved caspase-3 protein levels in Jurkat cells after irradiation (n=3). C-D. Representative Western blots and quantification showing cleaved caspase-3 protein levels in AC16 cells. CM was collected from Yoda1-treated BMDMs (Yoda1-BMDM-CM), Piezo1^fl/fl^ mouse BMDMs (Piezo1^fl/fl^-BMDM-CM) or Piezo1^Lyz2^ mouse BMDMs (Piezo1^Lyz2^-BMDM-CM). AC16 cells were cultured under glucose-oxygen deprivation (OGD) with fresh medium (FM) or CM (n=6). E-F. The brightfield images and viability of NRVMs under OGD with FM or CM from Yoda1-BMDMs, Piezo1^fl/fl^-BMDMs and Piezo1^Lyz2^-BMDMs (n=6). The data in B were analyzed via unpaired Student’s *t* test. The data in D and F were analyzed via one-way ANOVA, followed by the Bonferroni post hoc correction. FM, fresh medium. CM, conditioned medium.


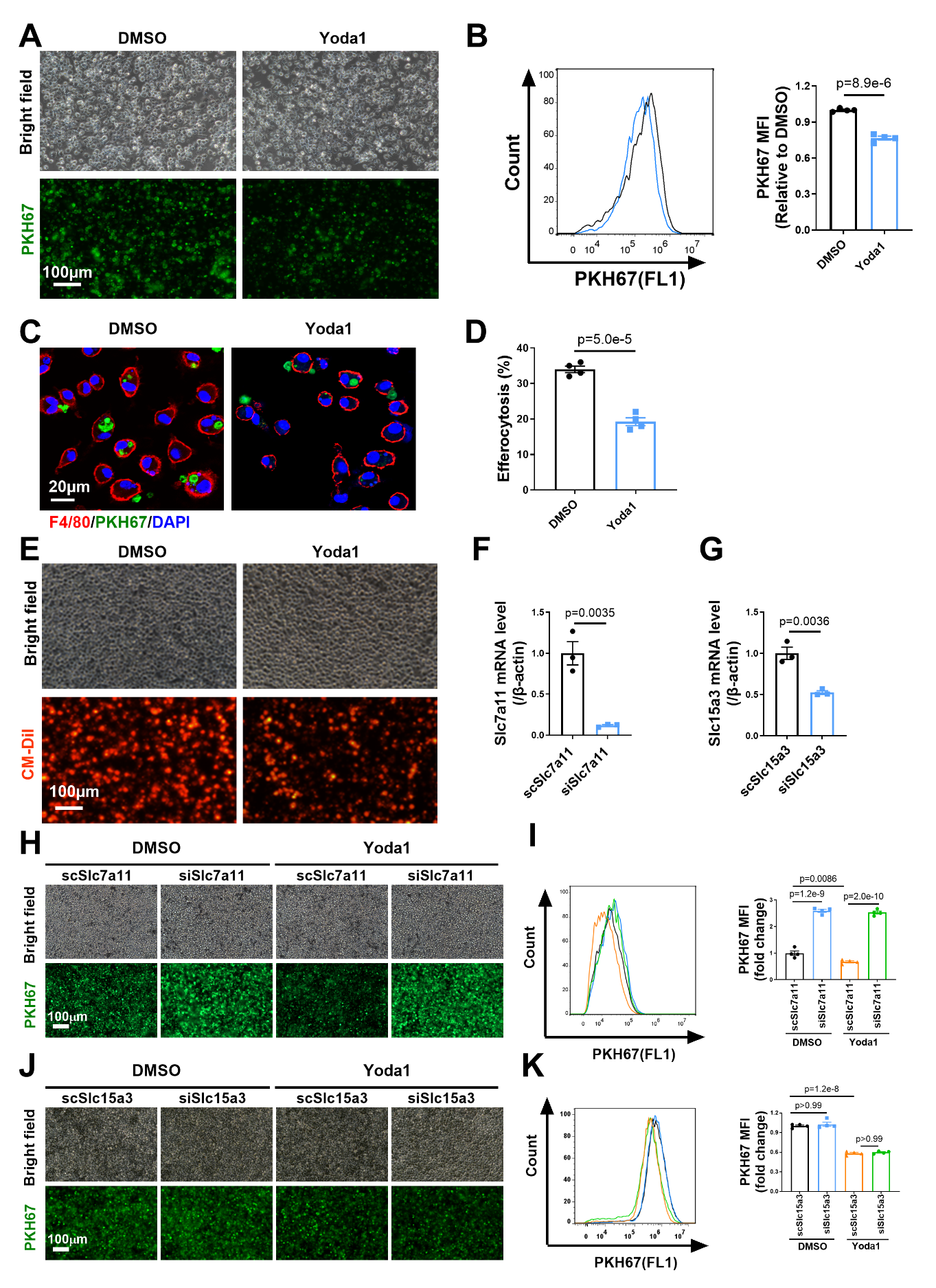


**Fig****ure S5**. **Piezo1 activation inhibited the efferocytotic activity of macrophages via SLC7A11 upregulation.** A-B. Fluorescence photographs (A) and flow cytometry results (B) revealed the uptake of PKH67-labeled apoptotic Jurkat cells by BMDMs (n=4). C-D. Confocal microscopy images and quantification of BMDMs that engulfed PKH67-labeled apoptotic Jurkat cells. BMDMs were stained with F4/80; BMDM nuclei were stained with 4′-6-diamidino-2-phenylindole (DAPI; blue) (n=4). E. Fluorescence images revealing the uptake of CM-DiI-labeled apoptotic Jurkat cells by PMs. From A through E, BMDMs and PMs were treated with 5 µM Yoda1 or DMSO for 12 h. F. The level of the Slc7a11 mRNA in BMDMs after control or SLC7A11 siRNA (siSlc7a11) transfection (n=3). G. The level of the Slc15a3 mRNA in BMDMs after control or SLC15A3 siRNA (siSlc15a3) transfection (n=3). H-I. Fluorescence images (H) and flow cytometry results (I) revealed the uptake of PKH67-labeled apoptotic Jurkat cells by BMDMs. BMDMs were transfected with scrambled RNA or SLC7A11 siRNA (siSlc7a11) and then treated with or without 5 µM Yoda1 for 12 h (n=4). J-K. Fluorescence images (J) and flow cytometry results (K) revealed the uptake of PKH67-labeled apoptotic Jurkat cells by BMDMs. BMDMs were transfected with scrambled RNA or SLC15A3 siRNA (siSlc15a3) and then treated with or without 5 µM Yoda1 for 12 h (n=4). The data in I and K were analyzed via one-way ANOVA, followed by the Bonferroni post hoc correction. Other data were analyzed via unpaired Student’s *t* test.


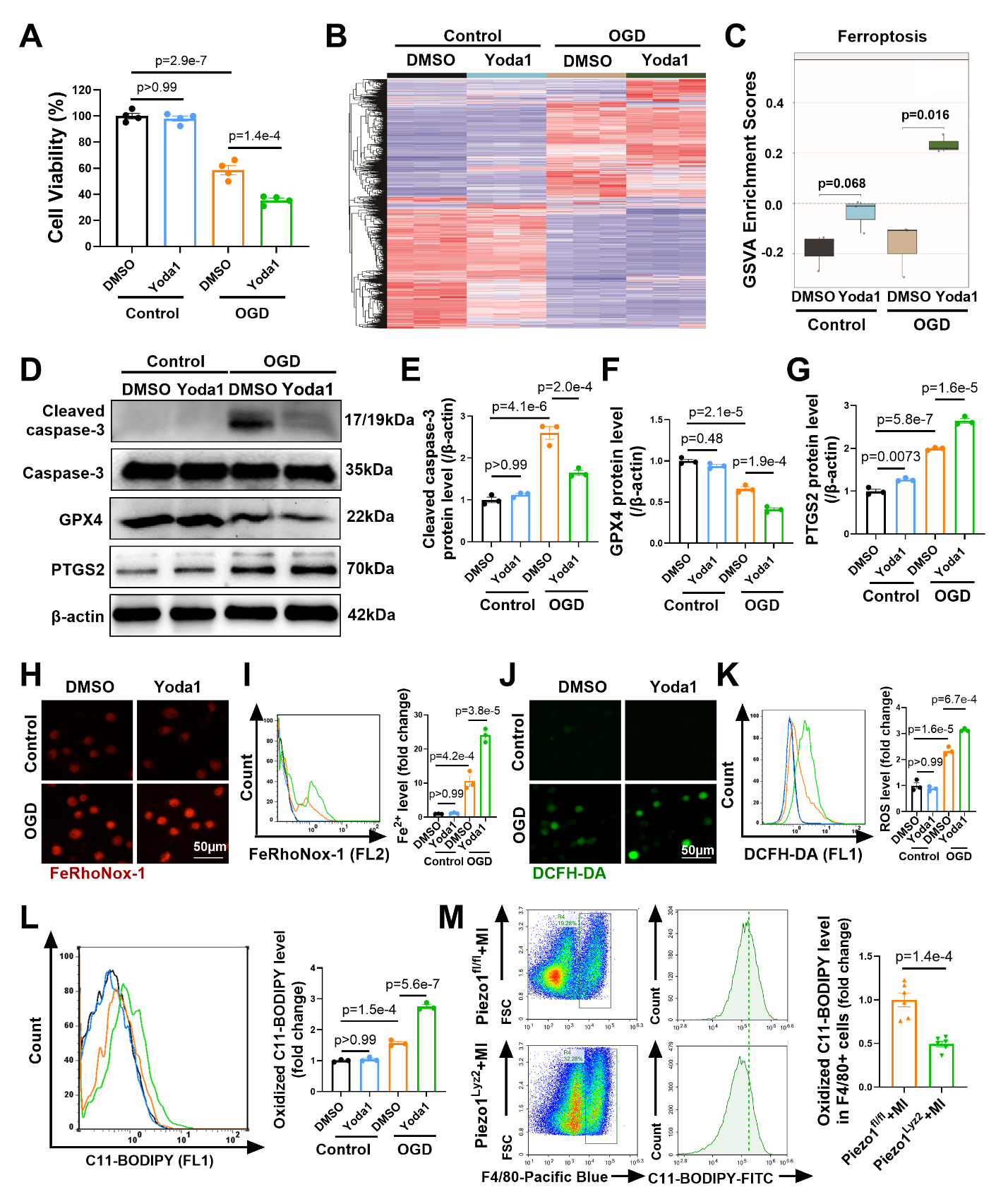


**Figure S6. Piezo1 exacerbated OGD-induced MoM ferroptosis.** A. Cell viability assays of BMDMs with a CCK-8 kit (n=4). BMDMs were treated with DMSO or Yoda1 (5 µM) under control or OGD for 9 h. B. Heatmap of the differentially expressed genes among 4 groups of macrophages. C. Ferroptosis scores calculated by gene set variation analysis (GSVA) between the Control+DMSO vs. Control+Yoda1 groups and the OGD+DMSO vs. OGD+Yoda1 groups. Data in C were analyzed by two-sided t-tests. P-values were adjusted for the false discovery rate (FDR) using the Benjamini-Hochberg method, with a significance level of α = 0.05. D-G. Representative Western blots and quantification showing cleaved caspase-3, GPX4 and PTGS2 protein levels in BMDMs treated with DMSO or Yoda1 (5 µM) under control or OGD for 9 h (n=3). H-I. The levels of Fe^2+^ in BMDMs were analyzed by immunostaining and flow cytometry analysis of FeRhoNox-1 (n=3). J-K. The levels of ROS in BMDMs were analyzed by immunostaining and flow cytometry analysis of DCFH-DA (n=3). L. The levels of lipid peroxidation in BMDMs were analyzed by flow cytometry analysis of oxidized C11-BODIPY signals (n=3). M. Flow cytometry analysis of macrophage lipid peroxidation by double staining of F4/80 and C11-BODIPY in cardiomyocyte-depleted cardiac cells in mice from Piezo1^fl/fl^+MI and Piezo1^Lyz2^+MI groups at day 7 (n=6 mice per group). The data in M were analyzed by unpaired Student’s *t* test. Other data were analyzed via one-way ANOVA, followed by the Bonferroni post hoc correction.


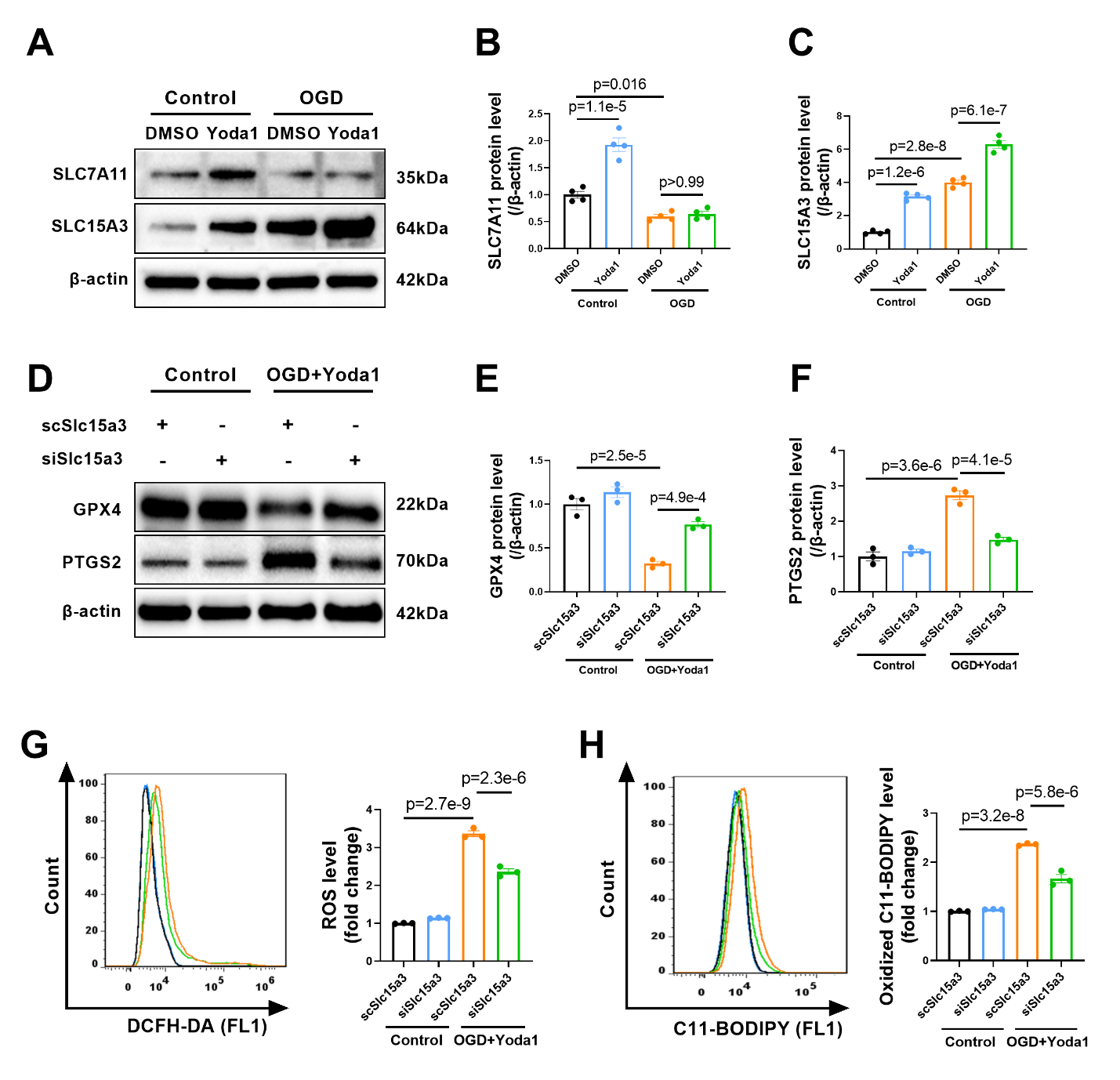


**Figure S7**. **Piezo1 exacerbated OGD-induced MoM ferroptosis by upregulating SLC15A3.**

A-C. Representative Western blots and quantification showing SLC7A11 and SLC15A3 protein levels in BMDMs (n=4). D-F. Representative Western blots and quantification of GPX4 and PTGS2 protein levels in BMDMs after control or Slc15a3 siRNA (siSlc15a3) transfection with or without OGD+Yoda1 treatment (n=3). G-H. Flow cytometry analysis of DCFH-DA (G) and oxidized C11-BODIPY (H) signals showing the levels of ROS and lipid peroxidation in BMDMs (n=3). Data were analyzed via one-way ANOVA, followed by the Bonferroni post hoc correction.


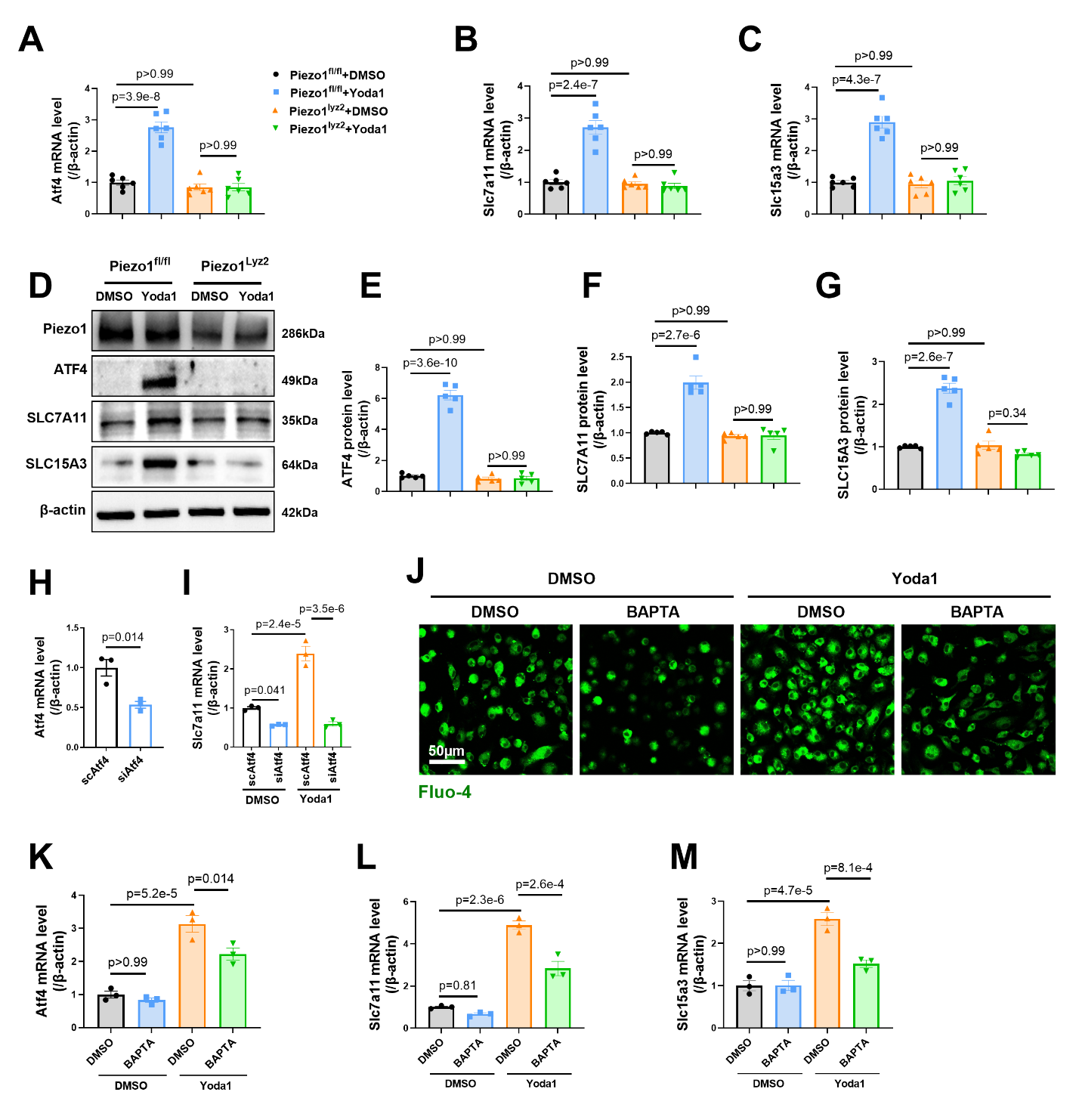


**Figure S8**. **Activation of Piezo1 upregulated ATF4/SLC7A11 and SLC15A3 via a Ca^2+^** **influx pathway.** A-C. The levels of the Atf4, Slc7a11 and Slc15a3 mRNAs in BMDMs from Piezo1^fl/fl^ and Piezo1^Lyz2^ mice that were treated with DMSO or Yoda1 (5 µM) for 12 h (n=6 mice per group). D-G. Representative Western blots and/or quantification showing the Piezo1, ATF4, SLC7A11 and SLC15A3 protein levels in BMDMs (n=5 mice per group). H. Atf4 mRNA expression in BMDMs after control or Atf4 siRNA transfection (n=3). I. The level of Slc7a11 mRNA in BMDMs after control or Atf4 siRNA transfection under DMSO or Yoda1 treatment (n=3). J. Fluo-4 fluorescence signals in BMDMs pretreated with 5 µM BAPTA-AM for 24 h, followed by DMSO or 5 µM Yoda1 treatment for 30 min were photographed using a confocal laser microscope. K-M. The levels of the Atf4, Slc7a11 and Slc15a3 mRNAs in BMDMs after DMSO or Yoda1 treatment with or without 5 µM BAPTA-AM pretreatment for 24 h (n=3). The data in A-G were analyzed via two-way ANOVA, followed by the Bonferroni post hoc correction. The data in H were analyzed via unpaired Student’s *t* test. Other data were analyzed via one-way ANOVA, followed by the Bonferroni post hoc correction.


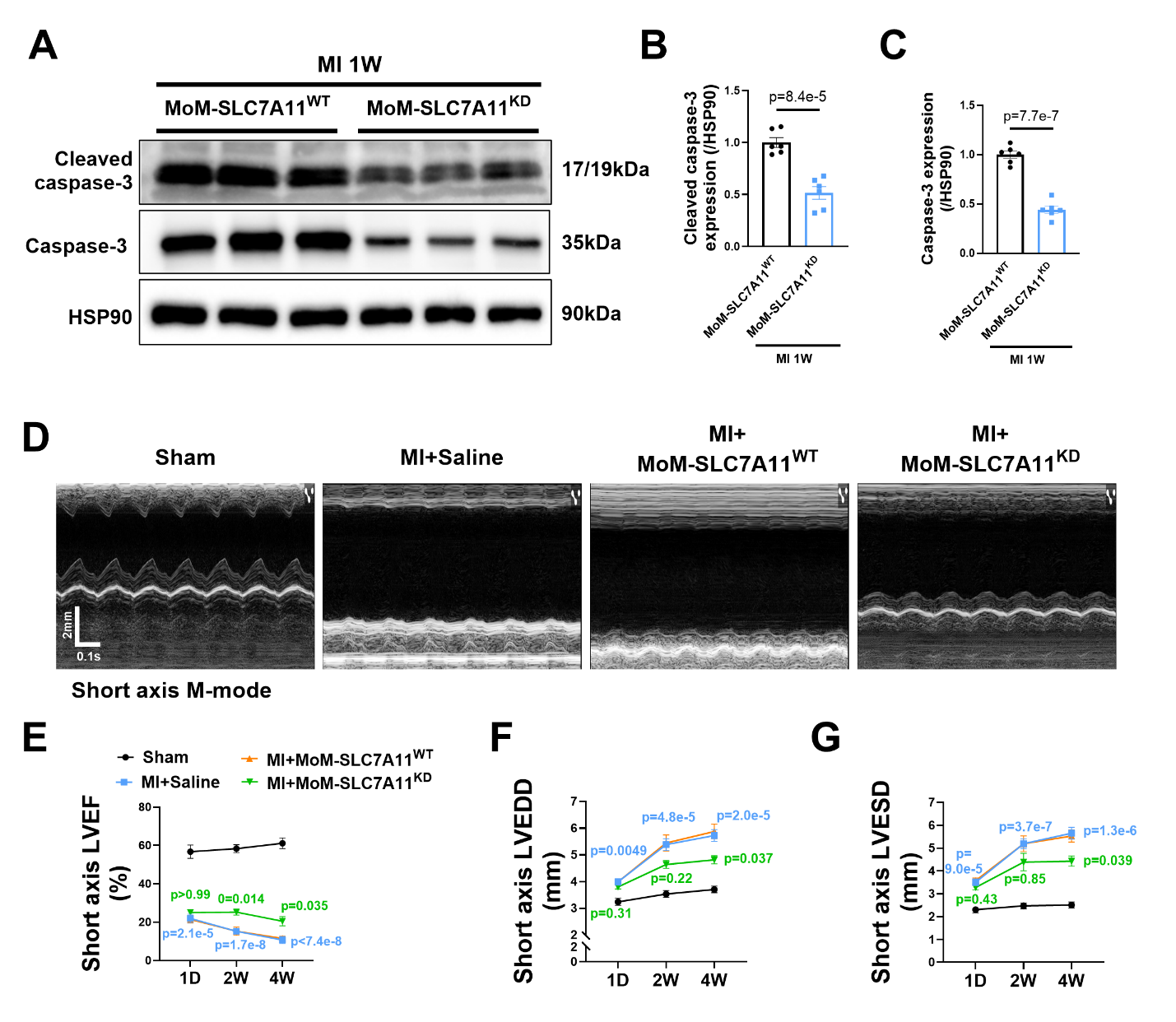


**Figure S9. BMDM-specific SLC7A11 knockdown reduced the number of apoptotic cardiomyocytes and ameliorated cardiac dysfunction after MI.** A-C. Representative Western blots and quantification showing caspase-3 and cleaved caspase-3 protein levels in heart tissues from MoM-SLC7A11^WT^ and MoM-SLC7A11^KD^ mice at 1 week after experimentally induced MI or the sham operation (n=6). D. Representative short-axis M-mode echocardiographic images at 4 weeks after MI. E‒G. The LVEF, LVEDD, and LVESD were evaluated via short-axis M-mode echocardiography (n= 7, 13, 12, and 12 at 1 d; n= 7, 9, 8, and 9 at 2 W; n= 7, 9, 8, and 9 at 4 W). Blue p compared with the sham group, and green p compared with the MI+MoM-SLC7A11^WT^ group. The data in B and C were analyzed by unpaired Student’s *t* test. The data in E-G were analyzed using a Mixed-effects model by Bonferroni's multiple comparisons test.

**Supplementary tables**

**Supplementary Table 1.** The clinical data of control and MI/R patients.

|  | non-MI (n = 10) | MI/R (n = 10) | p value |
| --- | --- | --- | --- |
| Age (y) | 58.2 ± 16.24 | 64.4 ± 15.714 | 0.40 |
| Male (n, %) | 6 (60) | 8 (80) | 0.33 |
| Hypertension history (n, %) | 6 (60) | 5 (50) | 0.65 |
| Diabetes history (n, %) | 4 (40) | 3 (30) | 0.64 |
| Brain disorder history (n, %) | 1 (10) | 2 (20) | 0.53 |
| Smoker (n, %) | 2 (20) | 4 (40) | 0.33 |
| Drinker (n, %) | 0 (0.0) | 3 (30) | 0.060 |
| cTNI (ng/ml) on admission | 0.0065 (0.00225~0.01) | 37.087 (10.332~106.966) | 1.5e-4 |
| Myoglobin (ng/ml) on admission | 26 (18.65~32.05) | 214.65 (45.725~387.025) | 8.8e-4 |
| CKMB (ng/ml) on admission | 1.5 (1.175~1.725) | 145.35(25.6~302.5) | 1.1e-3 |
| NT-proBNP (pg/ml) on admission | 52.775 (45.7175~217.4225) | 1462.5 (940.725~2677.25) | 2.9e-4 |
| cTNI (ng/ml) at discharge | 0.015 (0.00775~0.054) | 3.235 (1.21975~8.09675) | 1.6e-4 |
| Myoglobin (ng/ml) at discharge | 26.275 (21.0375~31.15) | 31.25 (21.475~36.9) | 0.45 |
| CKMB (ng/ml) at discharge | 1.665 (1.1525~1.85) | 3.8 (2.8~5.1) | 2.4e-4 |
| NT-proBNP (pg/ml) at discharge | 60.835 (50.815~401.075) | 1008.55 (423.625~1524.25) | 1.5e-3 |

cTNI, cardiac troponin I; CKMB, CreatineKinase-MB；NT-proBNP, N-Terminal Pro-Brain Natriuretic Peptide. The results are presented as the means ± SD, medians (interquartile ranges), or n (%). Differences between groups were analyzed by unpaired Student’s t test, the chi-square test, or the Mann‒Whitney U test.

**Supplementary Table 2.** Differentially expressed genes (mean count of >1000) between DMSO- and Yoda1-treated mouse BMDMs identified by RNA sequencing (RNA-seq) analysis and GO_BP analysis (34 total).

| **Gene ID** | **Fold Change（Yoda1/DMSO）** | **p value** | **Name** |
| --- | --- | --- | --- |
| ENSMUSG00000049723 | 3.966840438 | 2.24E-228 | Mmp12 |
| ENSMUSG00000027737 | 3.356760221 | 2.43E-19 | Slc7a11 |
| ENSMUSG00000029304 | 3.19482376 | 1.48E-34 | Spp1 |
| ENSMUSG00000021701 | 3.094314293 | 7.3E-115 | Plk2 |
| ENSMUSG00000022126 | 3.089224214 | 3.97E-53 | Acod1 |
| ENSMUSG00000051439 | 2.720345127 | 2.36E-115 | Cd14 |
| ENSMUSG00000025473 | 2.650337826 | 3.34E-115 | Adam8 |
| ENSMUSG00000029819 | 2.652448384 | 1.42E-55 | Npy |
| ENSMUSG00000042406 | 2.37222418 | 1.52E-61 | Atf4 |
| ENSMUSG00000024772 | 2.343254228 | 6.36E-31 | Ehd1 |
| ENSMUSG00000024737 | 2.317884239 | 1.3E-78 | Slc15a3 |
| ENSMUSG00000002944 | 2.237955117 | 8.23E-70 | Cd36 |
| ENSMUSG00000039191 | 2.21576041 | 1.01E-75 | Rbpj |
| ENSMUSG00000025044 | 2.20478552 | 1.26E-41 | Msr1 |
| ENSMUSG00000028691 | 2.182676157 | 1.24E-63 | Prdx1 |
| ENSMUSG00000020120 | 2.102668911 | 8.99E-65 | Plek |
| ENSMUSG00000030142 | 2.014685405 | 3.65E-17 | Clec4e |
| ENSMUSG00000031613 | 0.240893039 | 6.87E-30 | Hpgd |
| ENSMUSG00000038332 | 0.307112687 | 8.36E-109 | Sesn1 |
| ENSMUSG00000045322 | 0.343129766 | 3.3E-65 | Tlr9 |
| ENSMUSG00000049313 | 0.374820062 | 1.41E-89 | Sorl1 |
| ENSMUSG00000066800 | 0.389153049 | 7.58E-54 | Rnasel |
| ENSMUSG00000028859 | 0.40065827 | 6.52E-33 | Csf3r |
| ENSMUSG00000035493 | 0.402557647 | 8.22E-95 | Tgfbi |
| ENSMUSG00000040522 | 0.415960099 | 4.41E-53 | Tlr8 |
| ENSMUSG00000024610 | 0.431583889 | 7.73E-61 | Cd74 |
| ENSMUSG00000042286 | 0.432055194 | 6.24E-78 | Stab1 |
| ENSMUSG00000030830 | 0.44094682 | 2.87E-77 | Itgal |
| ENSMUSG00000035273 | 0.453613216 | 9.02E-72 | Hpse |
| ENSMUSG00000006611 | 0.458397216 | 6.71E-35 | Hfe |
| ENSMUSG00000044350 | 0.460220994 | 7.14E-38 | Lacc1 |
| ENSMUSG00000027111 | 0.464839887 | 4.55E-47 | Itga6 |
| ENSMUSG00000027009 | 0.468622851 | 1.74E-52 | Itga4 |
| ENSMUSG00000021127 | 0.470802305 | 1.28E-41 | Zfp36l1 |

**Supplementary Table 3. The sequences of the siRNAs.**

| **Genes** | **sense (5'—3')** | **antisense (5'—3')** |
| --- | --- | --- |
| Negative control | UUCUCCGAACGUGUCACGUTT | ACGUGACACGUUCGGAGAATT |
| Slc7a11 | CAAGUGGUUCAGACGAUUA | UAAUCGUCUGAACCACUUG |
| Slc15a3 | GAUCAGAUAGCAGUAACUA | UAGUUACUGCUAUCUGAUC |
| Atf4 | CUCUAGUCCAAGAGACUAA | UUAGUCUCUUGGACUAGAG |

**Supplementary Table 4. Real-time PCR primers.**

| **Genes** | **Species** | **Forward primer (5'—3')** | **Reverse primer (5'—3')** |
| --- | --- | --- | --- |
| Actin | Mouse | AACAGTCCGCCTAGAAGCAC | CGTTGACATCCGTAAAGACC |
| Piezo1 | Mouse | CACGGAACCTGACCTTGACA | ATCATGGCAATCAGGGCACA |
| IL-4 | Mouse | GGTCTCAACCCCCAGCTAGT | GCCGATGATCTCTCTCAAGTGAT |
| IL-6 | Mouse | TGGAGTACCATAGCTACCTGGA | TGGAAATTGGGGTAGGAAGGAC |
| CD36 | Mouse | ATGGGCTGTGATCGGAACTG | TTTGCCACGTCATCTGGGTTT |
| Stab1 | Mouse | TCAGCCACTCCAAATGAAGACT | CCAGTAAGCTCCACGCATTG |
| Trp53 | Mouse | TCCGAAGACTGGATGACTGC | GATCGTCCATGCAGTGAGGT |
| Atf4 | Mouse | TAAGTTGTGTGCTCGGGTGT | GATTTCGTGAAGAGCGCCA |
| Atf3 | Mouse | CGAGCGAAGACTGGAGCAAAA | GGATGGCGAATCTCAGCTCT |
| Nrf2 | Mouse | GACAAGAGCAACTCCAGAAGGAA | GAATGTGGGCAACCTGGGAGT |
| SLC7A11 | Mouse | TCTTCGATACAAACGCCCAGATA | GTGCTGAATGGGTCCGAGTAAAG |
| Col1a | Mouse | CTGGCGGTTCAGGTCCAAT | TCCAAACCACTGAAGCCTCG |
| Piezo2 | Mouse | AATCAAACCAACATTCCCCTTCA | CAGGTAGACGAGCAAAGGAGA |
| ANP | Mouse | GAAGATCCAGCTGCTTCGGG | CACACCACAAGGGCTTAGGA |
| BNP | Mouse | ATCTCAAGCTGCTTTGGGCA | CACTTCAAAGGTGGTCCCAGA |
| TNFa | Mouse | GTCCCCAAAGGGATGAGAAGT | TTTGCTACGACGTGGGCTAC |
| Il1b | Mouse | TGCCACCTTTTGACAGTGATG | TGATGTGCTGCTGCGAGATT |
| SLC15A3 | Mouse | TTTCCAGATGCAGTCCACCTATG | CTATCCGATCTCAGTGGCAAAGA |
| MMP12 | Mouse | CTGCTCCCATGAATGACAGTG | AGTTGCTTCTAGCCCAAAGAAC |
| SSP1 | Mouse | AGCAAGAAACTCTTCCAAGCAA | GTGAGATTCGTCAGATTCATCCG |
| PLK2 | Mouse | CCTGCGGACTATCACCTACCA | CTGCCCATCTTCAGAAGGCT |
| ACOD1 | Mouse | GGCACAGAAGTGTTCCATAAAGT | GAGGCAGGGCTTCCGATAG |
| CD14 | Mouse | ACTTCTCAGATCCGAAGCCAG | CCGCCGTACAATTCCACAT |
| ADAM8 | Mouse | TTGCCCCATGTGAAACAGTATG | AGGTGCAGGGTGAAAACGTG |
| NPY | Mouse | ATGCTAGGTAACAAGCGAATGG | CAGAATGCCCAAACACACGAG |
| END1 | Mouse | ACCACCGACTCCTTCATCG | AGCCGCAAAGTCATAACCTCG |
| RBPJ | Mouse | CTCCACCCAAACGACTCACTA | TCCAACCACTGCCCATAAGATA |
| MSR1 | Mouse | TGGAGGAGAGAATCGAAAGCA | CTGGACTGACGAAATCAAGGAA |
| PRDX1 | Mouse | AATGCAAAAATTGGGTATCCTGC | CGTGGGACACACAAAAGTAAAGT |
| PLEK | Mouse | CAAAGCGGATCAGGGAGGG | TTGACAAGGGCTAGTGAGAGT |
| CLEC4E | Mouse | AGTGCTCTCCTGGACGATAG | CCTGATGCCTCACTGTAGCAG |
| HPGD | Mouse | CAATGCAGGCGTGAACAATGA | GGTCCCACTGATAACAGAAACCA |
| SESN1 | Mouse | GGCCAGGACGAGGAACTTG | AAGGAGTCTGCAAATAACGCAT |
| TLR9 | Mouse | ATGGTTCTCCGTCGAAGGACT | GAGGCTTCAGCTCACAGGG |
| SORL1 | Mouse | GAACGGAGTGTGCATCAGCTT | GCAGCGGAAGTAATTGGGC |
| RNASEL | Mouse | TAGGCGAACACATCAATGAGGA | CTGCCTCTGGAACGCTGAG |
| CSF3R | Mouse | CTGATCTTCTTGCTACTCCCCA | GGTGTAGTTCAAGTGAGGCAG |
| TGFBI | Mouse | CAGCACGGCCCCAATGTAT | GGGACCTTTTCATATCCAGGACA |
| TLR8 | Mouse | GAAAACATGCCCCCTCAGTCA | CGTCACAAGGATAGCTTCTGGAA |
| CD74 | Mouse | CCGCCTAGACAAGCTGACC | ACAGGTTTGGCAGATTTCGGA |
| ITGAL | Mouse | TGGCAACAGACATACACCTACG | GGTGGTAGTCTTCTTGAGTGTG |
| HPSE | Mouse | ACCGACGACGTGGTAGACTT | GCAGGAGATAAGCCTCTAGCC |
| HFE | Mouse | CACCGCGTTCACATTCTCTAA | CTGGCTTGAGGTTTGCTCC |
| LACC1 | Mouse | ACTCCGGCTGGAAAGGTACAT | TGACGGTCCCAGAACAACAAT |
| LTGA6 | Mouse | TGCAGAGGGCGAACAGAAC | GCACACGTCACCACTTTGC |
| Itga4 | Mouse | AACCGGGCACTCCTACAAC | CACCACCGAGTAGCCAAACAG |
| ZFP36l1 | Mouse | GCTTTCGAGACCGCTCTTTCT | TTGTCCCCGTACTTACAGGCA |
| β-actin | Human | CATGTACGTTGCTATCCAGGC | CTCCTTAATGTCACGCACGAT |
| Piezo1 | Human | GGACTCTCGCTGGTCTACCT | GGGCACAATATGCAGGCAGA |
