# Supplementary figures and images for: Piezo1 Upregulation in Monocyte‐Derived Macrophages Impairs Post‐Myocardial Infarction Cardiac Repair via Defective Efferocytosis and Enhanced Ferroptosis
Source: Adv Sci (Weinh). 2025 Nov 10;13(5):e10991. doi: 10.1002/advs.202510991 (PMC12850066; doi:10.1002/advs.202510991)

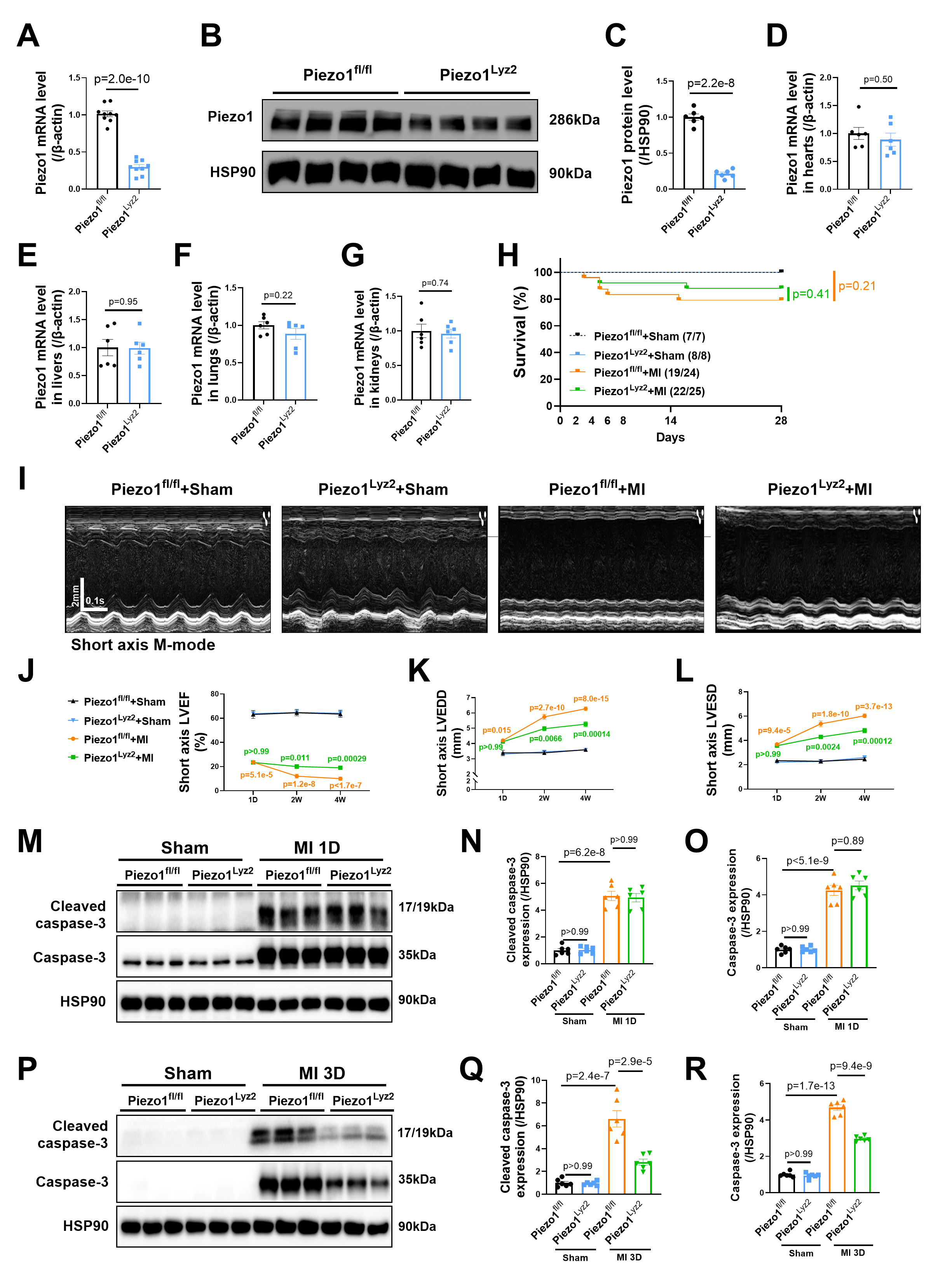

Supplement: Supplementary file 2 — Supporting Information [file ADVS-13-e10991-s002.zip › Online Figure 3.tif]

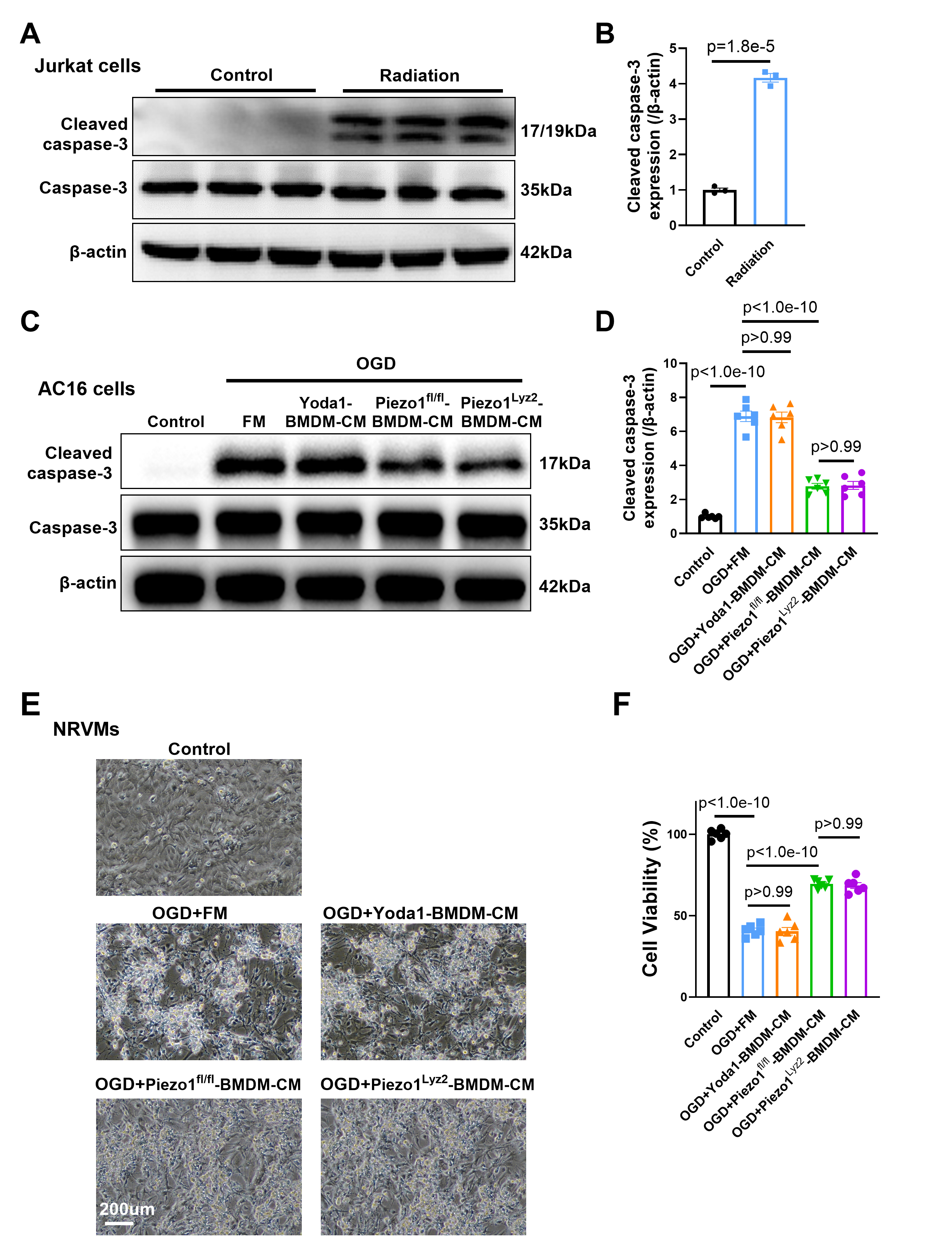

Supplement: Supplementary file 2 — Supporting Information [file ADVS-13-e10991-s002.zip › Online figure 4.tif]

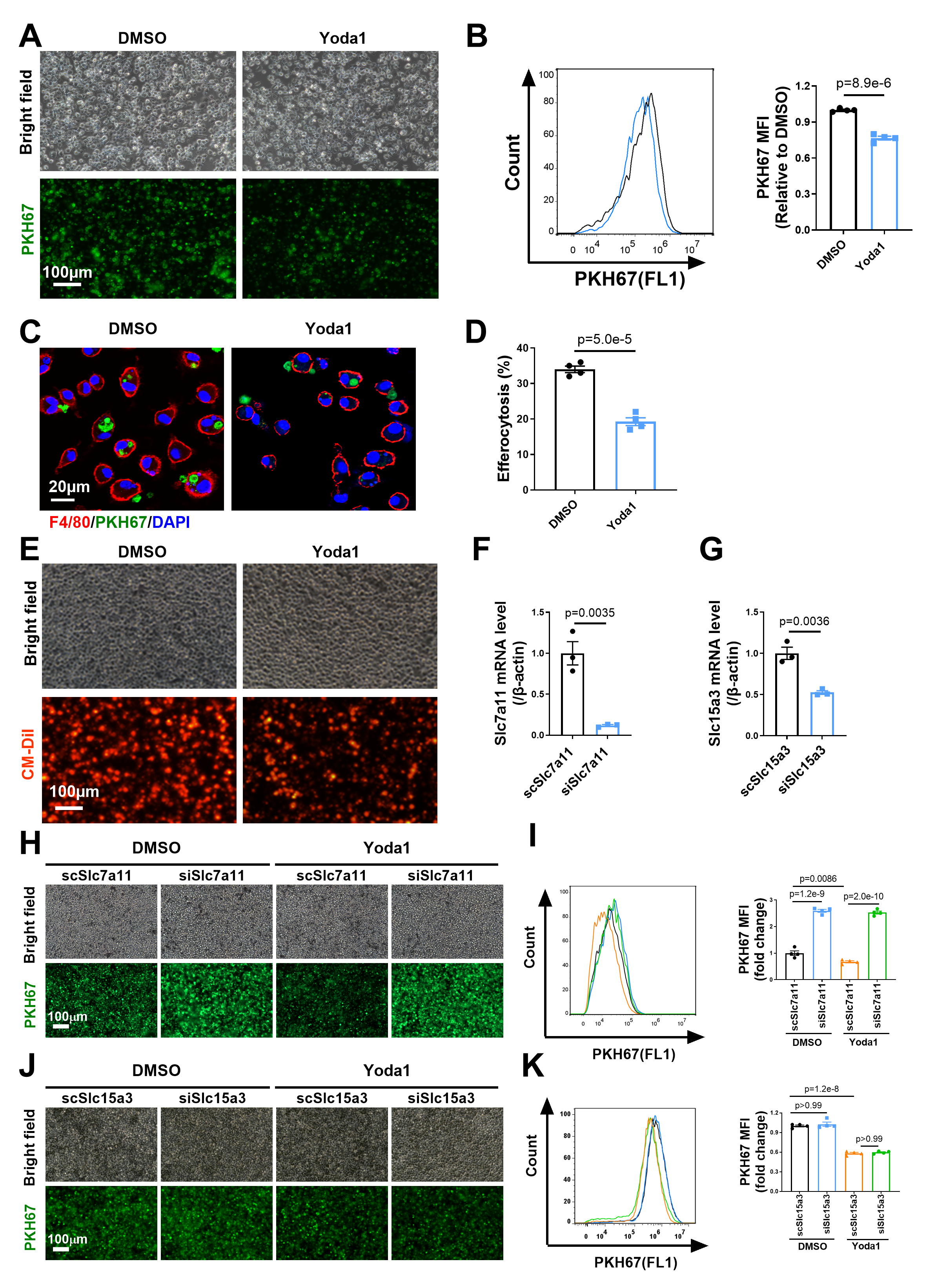

Supplement: Supplementary file 2 — Supporting Information [file ADVS-13-e10991-s002.zip › Online Figure 5.tif]

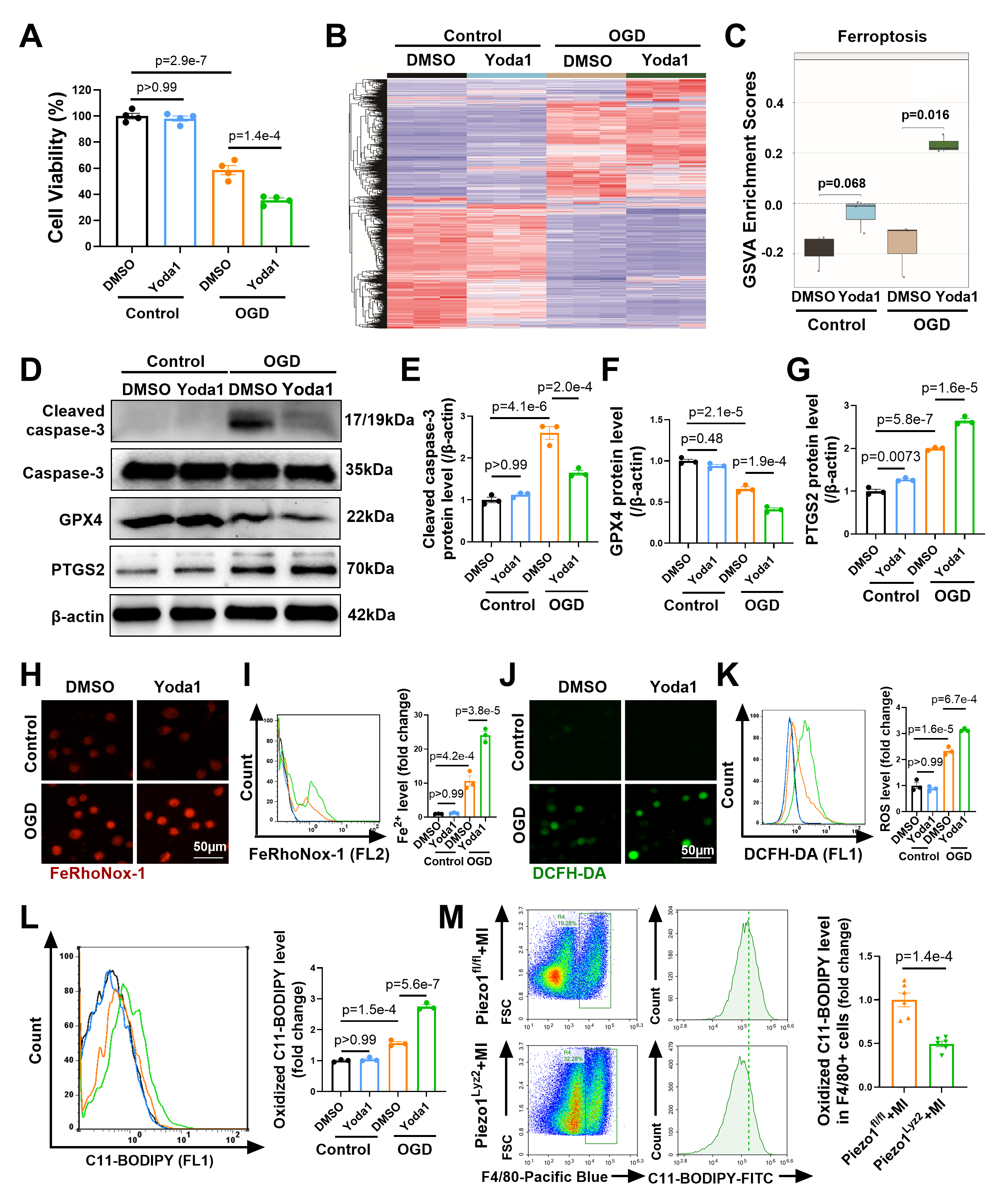

Supplement: Supplementary file 2 — Supporting Information [file ADVS-13-e10991-s002.zip › Online Figure 6.tif]

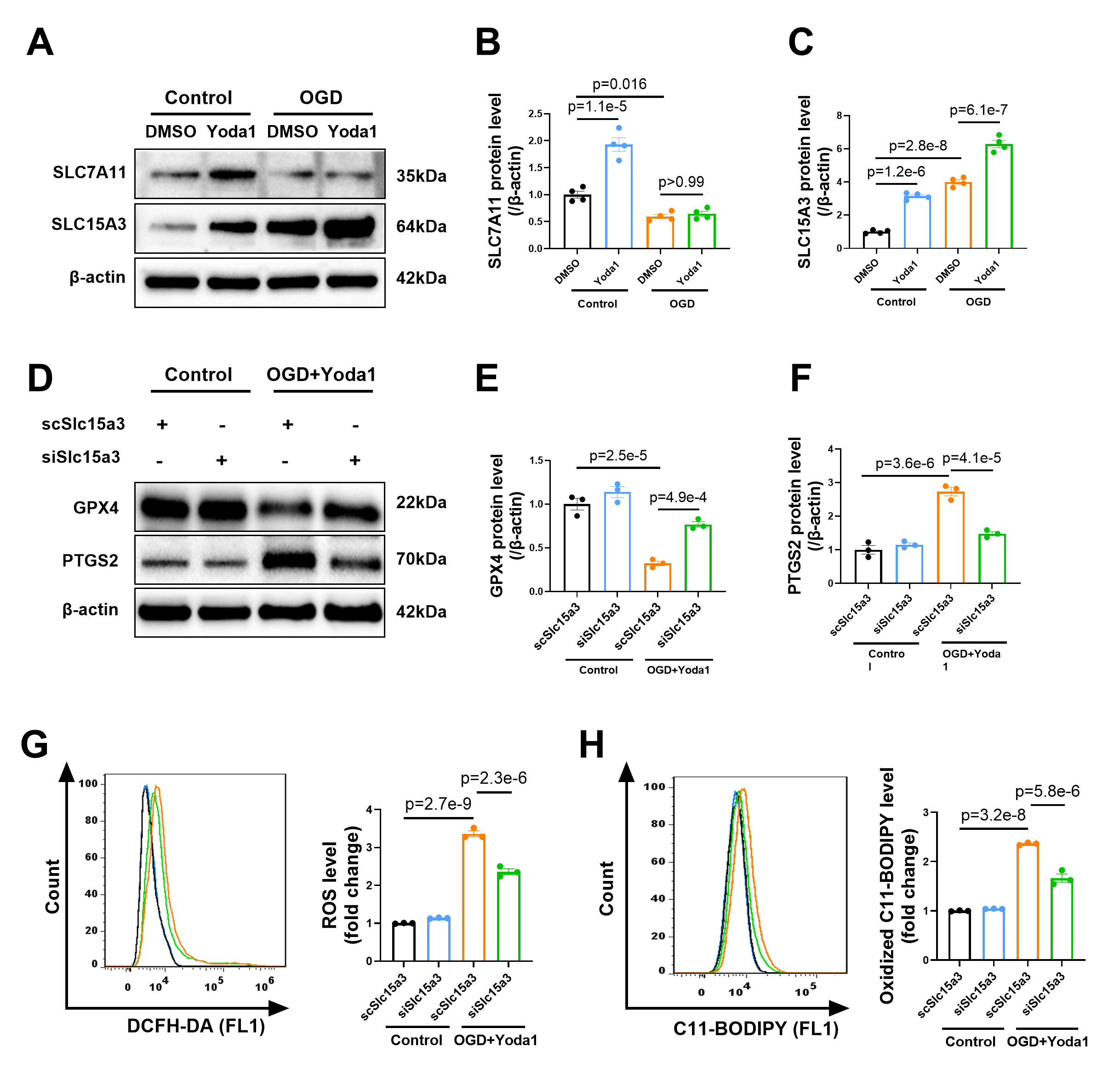

Supplement: Supplementary file 2 — Supporting Information [file ADVS-13-e10991-s002.zip › Online Figure 7.tif]

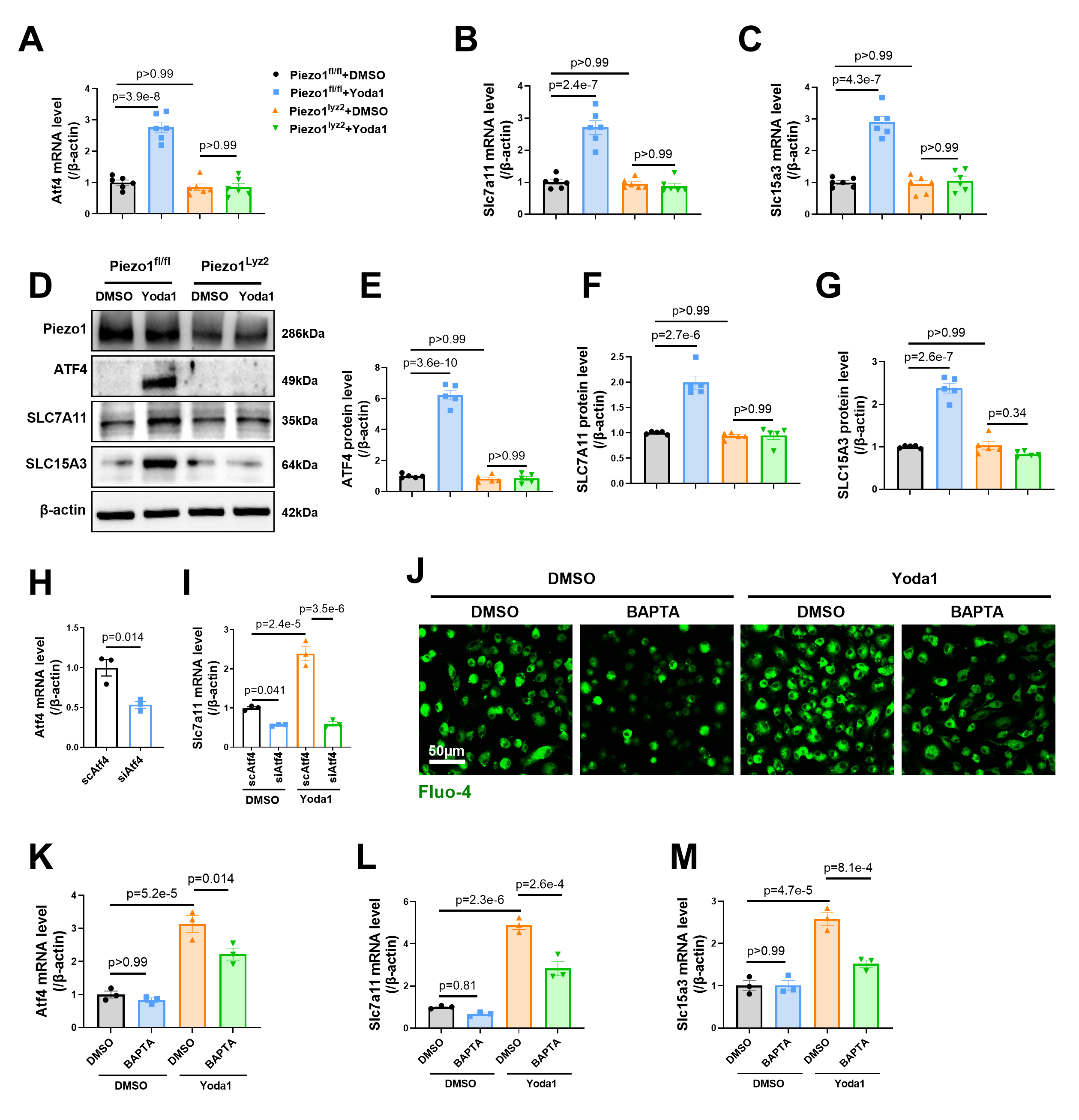

Supplement: Supplementary file 2 — Supporting Information [file ADVS-13-e10991-s002.zip › Online Figure 8.tif]

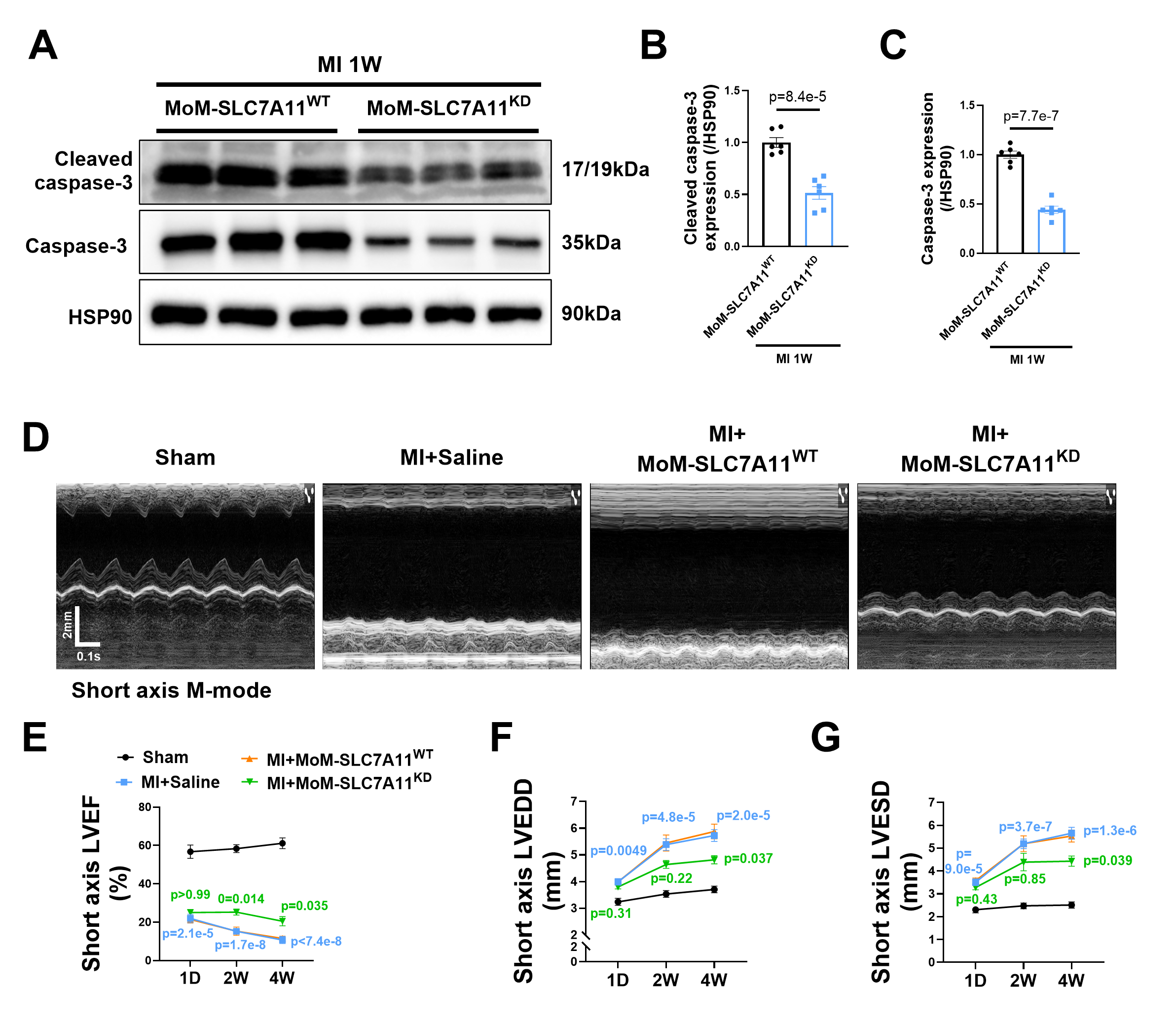

Supplement: Supplementary file 2 — Supporting Information [file ADVS-13-e10991-s002.zip › Online Figure 9.tif]

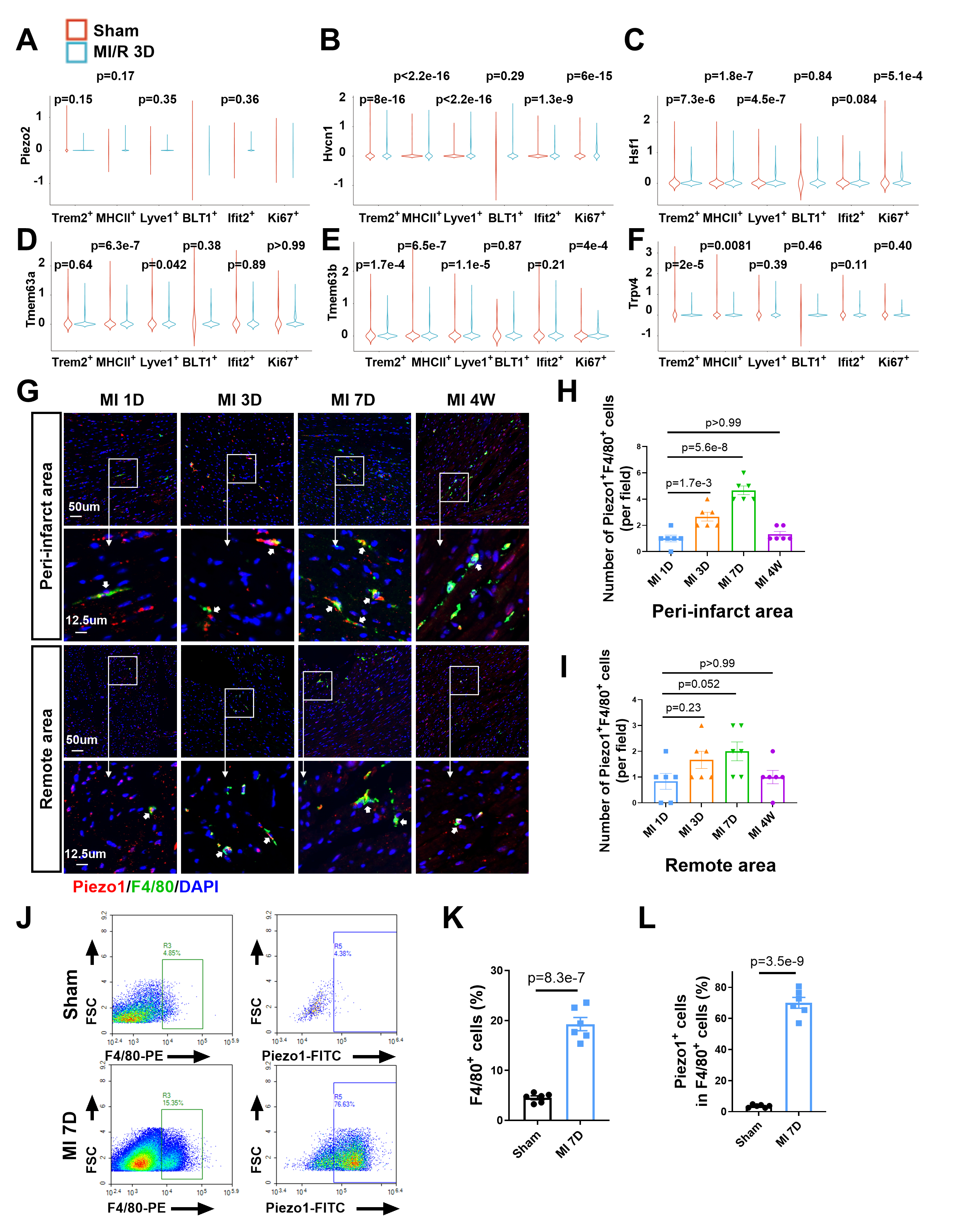

Supplement: Supplementary file 2 — Supporting Information [file ADVS-13-e10991-s002.zip › Online figure 1.tif]

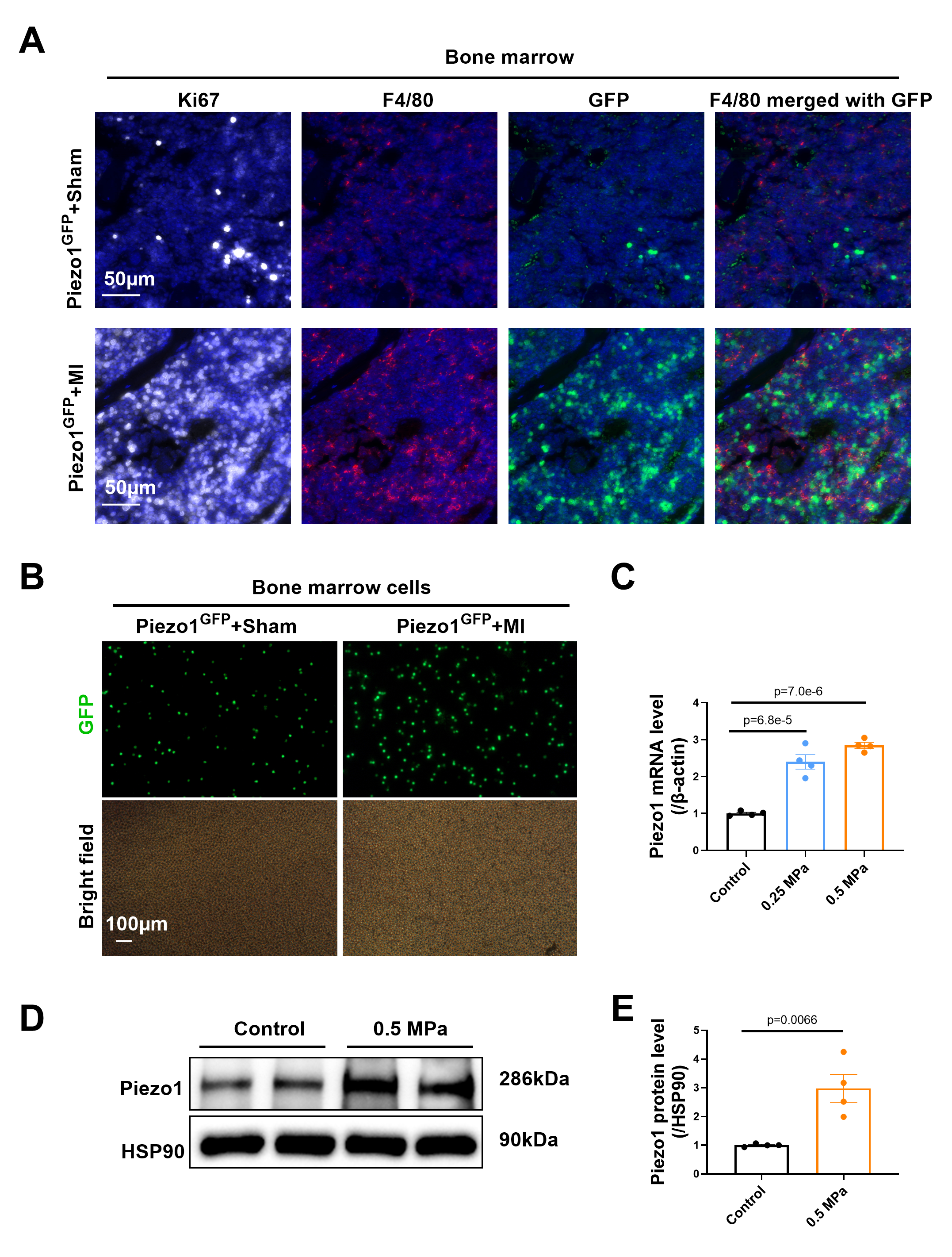

Supplement: Supplementary file 2 — Supporting Information [file ADVS-13-e10991-s002.zip › Online Figure 2.tif]
